# RBBP6 maintains glioblastoma stem cells through CPSF3-dependent alternative polyadenylation

**DOI:** 10.1038/s41421-024-00654-3

**Published:** 2024-03-19

**Authors:** Peng Lin, Wenyan Chen, Zhilin Long, Jichuan Yu, Jiayao Yang, Zhen Xia, Qiulian Wu, Xinyu Min, Jing Tang, Ya Cui, Fuyi Liu, Chun Wang, Jian Zheng, Wei Li, Jeremy N. Rich, Lei Li, Qi Xie

**Affiliations:** 1 College of Life Sciences, Zhejiang University, Hangzhou, Zhejiang China; 2 Westlake Disease Modeling Laboratory, Westlake Laboratory of Life Sciences and Biomedicine, Hangzhou, Zhejiang China; 3 Key Laboratory of Growth Regulation and Translational Research of Zhejiang Province, School of Life Sciences, Westlake University, Hangzhou, Zhejiang China; 4 Shenzhen Bay Laboratory, Shenzhen, Guangdong China; 5 University of Pittsburgh Medical Center Hillman Cancer Center, Department of Neurology, University of Pittsburgh, Pittsburgh, PA USA; 6 Division of Computational Biomedicine, Department of Biological Chemistry, School of Medicine, University of California, Irvine, Irvine, CA USA; 7 Department of Neurosurgery, The Second Affiliated Hospital of Zhejiang University School of Medicine, Hangzhou, Zhejiang China

**Keywords:** CNS cancer, Ubiquitylation, Mechanisms of disease, miRNAs, Cancer stem cells

## Abstract

Glioblastoma is one of the most lethal malignant cancers, displaying striking intratumor heterogeneity, with glioblastoma stem cells (GSCs) contributing to tumorigenesis and therapeutic resistance. Pharmacologic modulators of ubiquitin ligases and deubiquitinases are under development for cancer and other diseases. Here, we performed parallel in vitro and in vivo CRISPR/Cas9 knockout screens targeting human ubiquitin E3 ligases and deubiquitinases, revealing the E3 ligase RBBP6 as an essential factor for GSC maintenance. Targeting RBBP6 inhibited GSC proliferation and tumor initiation. Mechanistically, RBBP6 mediated K63-linked ubiquitination of Cleavage and Polyadenylation Specific Factor 3 (CPSF3), which stabilized CPSF3 to regulate alternative polyadenylation events. RBBP6 depletion induced shortening of the 3’UTRs of MYC competing-endogenous RNAs to release miR-590-3p from shortened UTRs, thereby decreasing MYC expression. Targeting CPSF3 with a small molecular inhibitor (JTE-607) reduces GSC viability and inhibits in vivo tumor growth. Collectively, RBBP6 maintains high MYC expression in GSCs through regulation of CPSF3-dependent alternative polyadenylation, providing a potential therapeutic paradigm for glioblastoma.

## Introduction

Glioblastoma is the most prevalent and lethal primary brain tumor, with a median survival time of less than two years. Current standard-of-care for glioblastoma includes maximal surgical resection, combined radiotherapy and chemotherapy, followed by adjuvant chemotherapy, offering only palliation^1^. Glioblastoma displays intratumoral heterogeneity with self-renewing glioblastoma stem cells (GSCs) at the hierarchical apex^2^. GSCs are functionally characterized by their capacities of self-renewal and tumor initiation with additional contributions to tumor angiogenesis, radio-resistance, and chemoresistance^3^.

Glioblastomas were one of the first cancers to under comprehensive genetic analysis, which revealed frequent alterations leading to dysregulated signaling, including those involving epigenetic alterations, transcriptional regulators, and posttranscriptional regulation^3–5^. However, precision medicine based on genetic lesions has had a limited impact on the clinical management of glioblastoma patients, suggesting that alternative molecular targets may offer therapeutic benefits. Ubiquitination is one of the most common post-translational modifications that regulate the fate and/or function of substrate proteins. The ubiquitin‒proteasome pathway maintains the homeostasis of most proteins^6,7^. Ubiquitin molecules are added to target proteins through a catalytic cascade involving E1 (ubiquitin-activating), E2 (ubiquitin-conjugating), and E3 (ubiquitin ligase) enzymes, which can be reversed by deubiquitinating enzymes (DUBs)^8^. Prior studies have linked ubiquitin pathways to glioblastoma growth, in general, and GSC maintenance, specifically. For example, glioblastomas can overexpress USP15, which binds to the SMAD7-SMAD specific E3 ubiquitin protein ligase 2 (SMURF2) complex to deubiquitinate and stabilize the type I TGF-β receptor (TβR-I), enhancing TGF-β signaling^9^. In GSCs, USP9x abrogates aldehyde dehydrogenase 1A3 (ALDH1A3) polyubiquitination to stabilize ALDH1A3 and maintain GSCs^10^. Treatment of GSCs with the USP9x inhibitor WP1130 induces ALDH1A3 degradation and inhibits GSC growth^10^. Further, USP13 and FBXL14 reciprocally modulate MYC polyubiquitination in GSCs^11^. Therefore, deubiquitinases and ubiquitin ligases may be potential therapeutic targets in glioblastoma.

CRISPR/Cas9-based knockout (KO) high-throughput screening has been proven to be an effective platform for the discovery of target genes in cancer therapy. Previous studies of CRISPR screens in glioblastoma have largely focused on in vitro cultured cell models that do not adequately represent the complex effects of the tumor microenvironment, an approach that may limit the success of drug development. Here, we adapted a more physiologically relevant in vivo CRISPR screen to identify essential ubiquitin ligases and deubiquitinases for GSC maintenance and tumor formation.

## Results

### In vitro and in vivo CRISPR screens revealed novel regulators of GSC-derived tumor growth

**Fig. 1:**
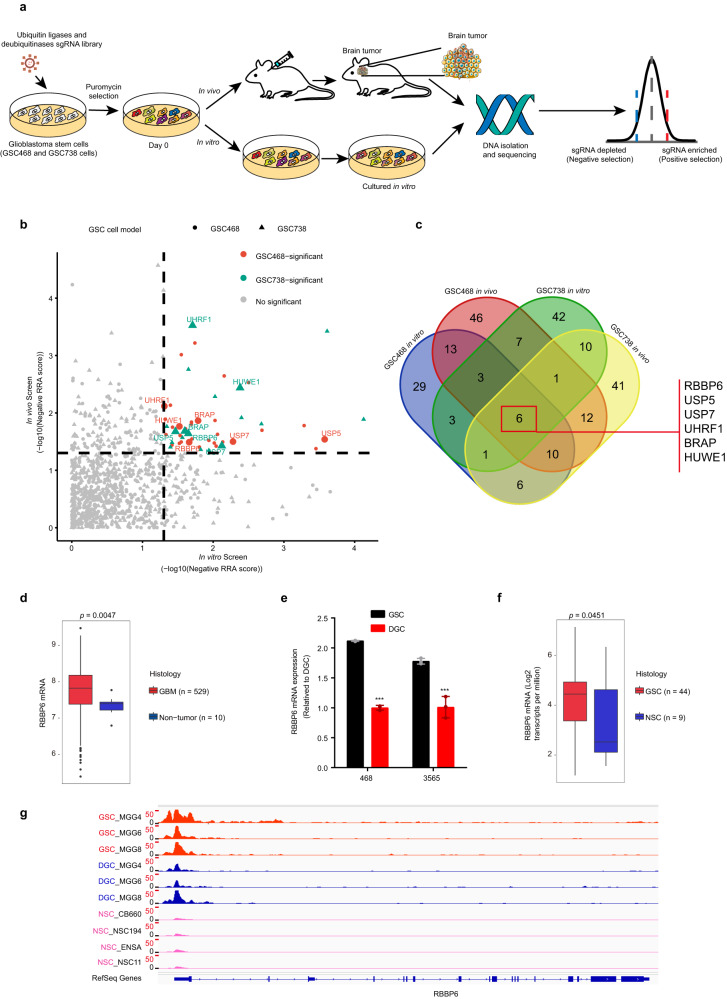
CRISPR/Cas9 screens revealed novel regulators of GSCs. **a** Workflow of the CRISPR screens to identify potential targets for glioblastoma therapy. **b** Dot plot represents the CRISPR screen results. **c** Venn diagram showing the overlap between the in vivo CRISPR screen and in vitro CRISPR screen results in GSCs. **d** *RBBP6* mRNA expression levels in the TCGA GBM dataset (U133A). Statistical significance was assessed using a *t*-test. **e** The mRNA level of *RBBP6* in GSCs and matched DGCs. Statistical significance was assessed using a *t*-test, ***P < 0.001, *n* = 3. **f** *RBBP6* mRNA expression level in a panel of 44 GSCs and 9 NSCs. Statistical significance was assessed using a *t*-test. **g** H3K27ac signal at the *RBBP6* locus in three GSCs, three DGCs and four NSCs.

To systematically interrogate the functional contributions of DUBs and ubiquitin ligases to GSC, we performed parallel in vitro and in vivo CRISPR screens to identify DUBs and ubiquitin ligases essential for GSC survival and tumor formation. We designed and constructed a lentivirus-based CRISPR/Cas9 KO library targeting 387 human ubiquitin ligases and 76 deubiquitinases. The library contained 8 different sgRNAs for each targeted gene and 100 nontargeting, control sgRNAs (Supplementary Table S1). We then lentivirally transduced the library into two patient-derived GSCs (GSC468 and GSC738) and then performed puromycin selection. GSCs with CRISPR/Cas9-mediated KO were split into two groups: (1) in vitro cultures in stem cell conditions, and (2) an in vivo screening group intracranially implanted into immunocompromised NSG (NOD.Cg-Prkdc scid Il2rg tm1Wjl /SzJ) mice (Fig. 1a). We analyzed changes in sgRNA abundance in vivo and in vitro using MAGeCK, a bioinformatics pipeline for analysis of pooled genetic screening data. To identify potential therapeutic targets in glioblastoma, we focused on the depleted sgRNAs in our CRISPR screen results. Thirteen genes were hit common to both GSCs in vitro, while 29 genes were hit common to both GSCs in vivo. We next identified 6 highly confident hits common to both the in vitro and in vivo screens. These 6 hits included: RB Binding Protein 6, Ubiquitin Ligase (RBBP6), Ubiquitin Specific Peptidase 5 (USP5), Ubiquitin Specific Peptidase 7 (USP7), Ubiquitin Like with PHD And Ring Finger Domains 1 (UHRF1), BRCA1 Associated Protein (BRAP), and HECT, UBA And WWE Domain Containing E3 Ubiquitin Protein Ligase 1 (HUWE1) (Fig. 1b, c).

To prioritize hits for further study, we examined the expression of these genes in The Cancer Genome Atlas (TCGA)-glioblastoma dataset. Of the 6 shared genes, only the mRNA expression of RBBP6 was upregulated in glioblastoma tissue compared to normal brain tissue (Fig. 1d; Supplementary Fig. S1a). Furthermore, RNA sequencing (RNA-seq) and H3K27ac (Histone 3 lysine 27 acetyl, a marker of active enhancer and promoter regions) chromatin immunoprecipitation followed by deep sequencing (ChIP-seq) data from our group and other published datasets^12,13^ showed that RBBP6 was preferentially expressed in GSCs relative to normal neural stem cells (NSCs) and differentiated glioblastoma cells (DGCs) (Fig. 1e–g). Therefore, we selected RBBP6 for further study.

### RBBP6 maintained GSCs

**Fig. 2:**
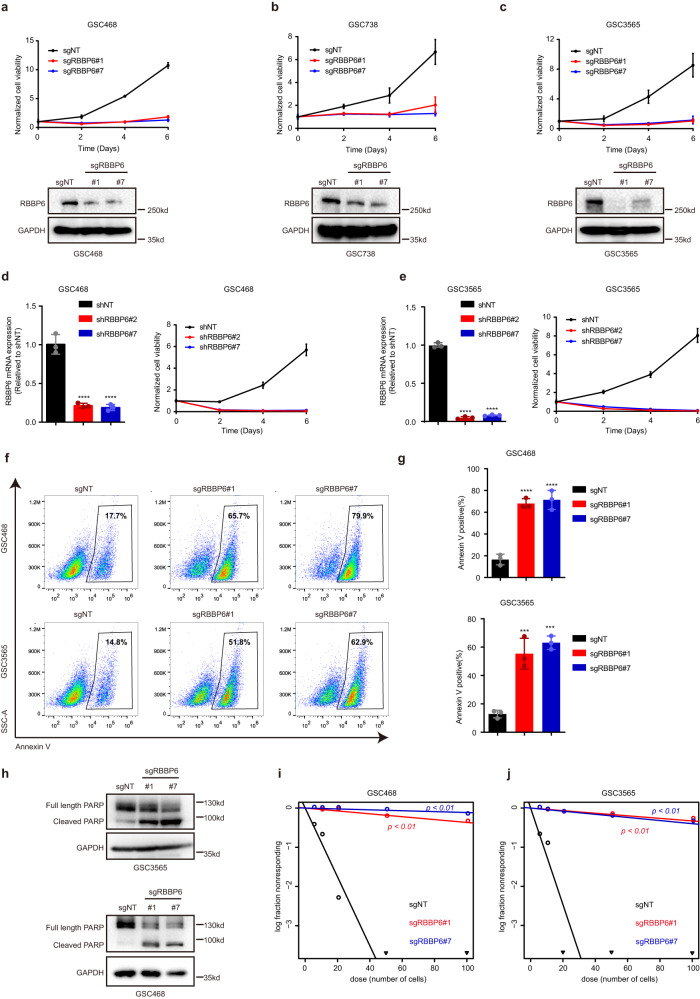
RBBP6 maintained GSCs. **a**–**c** Cell viability in the GSC468 cell model (**a**), GSC738 cell model (**b**), and GSC3565 cell model (**c**) transduced with two separate sgRNAs targeting *RBBP6* or sgNT. Three technical replicates were used for each group (Top). The error bars show the standard deviations (SDs). Western blot results from the Top assays (Bottom). **d**, **e** The mRNA level of *RBBP6* in GSC468 (**d**) and GSC3565 (**e**) cells transduced with two separate shRNAs targeting *RBBP6* or shNT (Left). Statistical significance was assessed using an ordinary one-way ANOVA with Dunnett’s multiple comparisons test, ****P < 0.0001, *n* = 3. Cell viability in the GSC468 cell model (**d**) and GSC3565 cell model (**e**) transduced with two separate shRNAs targeting *RBBP6* or shNT (Right). Three technical replicates were used for each group. The error bars show the SDs. **f**, **g** Annexin V staining of GSC3565 and GSC468 cells transduced with two separate sgRNAs targeting *RBBP6* or sgNT. Quantification of Annexin-V staining using an ordinary one-way ANOVA with Dunnett’s multiple comparisons test, ***P < 0.001, ****P < 0.0001, *n* = 3. **h** Western blot of PARP in GSC468 and GSC3565 cells transduced with two separate sgRNAs targeting *RBBP6* or sgNT. **i**, **j** The tumor sphere formation capacity was measured in vitro by ELDAs in GSC468 and GSC3565 cells transduced with two separate sgRNAs targeting *RBBP6* or sgNT.

To investigate the role of RBBP6 in GSCs, we performed *RBBP6* KO with two independent sgRNAs in 3 patient-derived GSCs (GSC468, GSC738, and GSC3565) to confirm the CRISPR screen results. *RBBP6* KO inhibited cell proliferation compared to cells transduced with a sgRNA control (Fig. 2a–c). We confirmed this phenotype in orthogonal studies by applying 2 nonoverlapping shRNAs to knock down *RBBP6* mRNA expression and confirmed that RBBP6 inhibition impaired GSC proliferation (Fig. 2d, e). A similar loss of growth was detected upon RBBP6 knockdown in two patient-derived primary cells (Supplementary Fig. S2a–d). *RBBP6* KO induced apoptosis, as measured by annexin V staining and PARP cleavage in GSCs but not non-stem tumor cells (NSTCs) and iPSC-induced astrocytes and neural progenitor cells (NPCs) (Fig. 2f–h; Supplementary Fig. S2e–h). Extreme limiting dilution assays (ELDAs) demonstrated that depletion of RBBP6 decreased both the frequency and self-renewal of GSCs (Fig. 2i, j). Taken together, these findings indicate that RBBP6 is essential for GSC maintenance.

### RBBP6 depletion decreased MYC expression and downstream MYC effectors

**Fig. 3:**
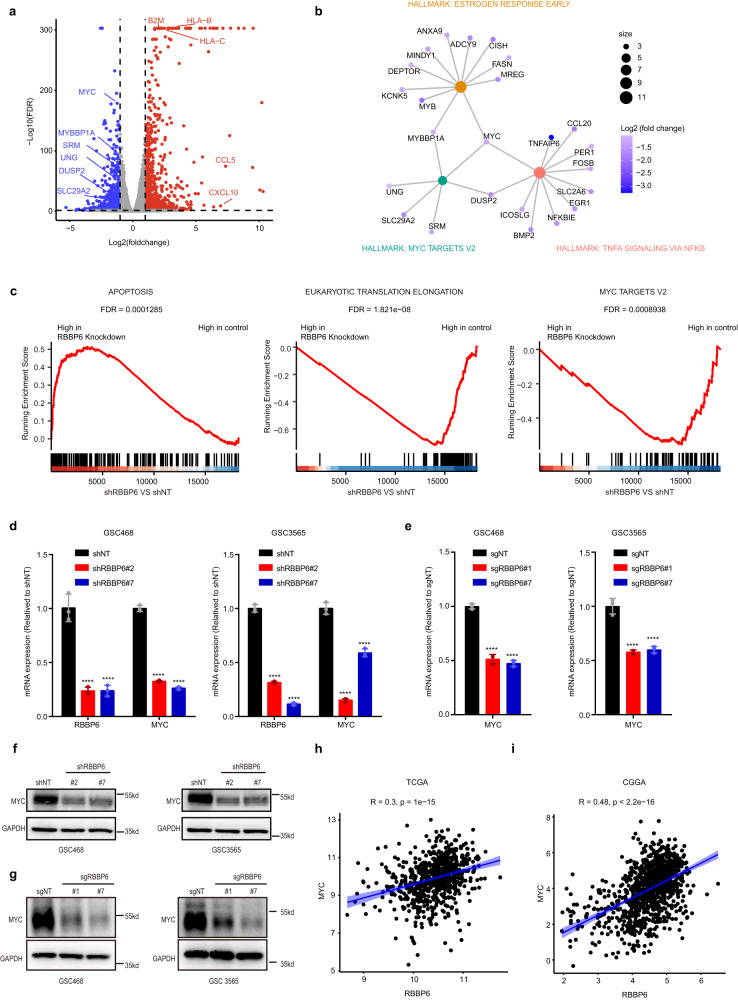
RBBP6 depletion downregulated MYC expression. **a** Volcano plot showing the fold changes in the normalized RNA read counts (shRBBP6#2 vs shNT) on the *x*-axis (Log_2_) and the FDR values on the *y*-axis (–Log_10_). The blue dots indicate significantly downregulated genes and the red dots indicate upregulated genes in GSC468 *shRBBP6* cells compared with GSC468 shNT cells (FDR ≤ 0.05 and Log_2_FoldChange difference ≥ 1). **b** Hallmark enrichment analysis of downregulated genes following RBBP6 knockdown. **c** GSEA of differentially expressed genes (DEGs) in RBBP6 knockdown and control GSC468 cells. **d** mRNA levels of *RBBP6* and *MYC* in GSC468 (left) and GSC3565 (right) cell models transduced with two separate shRNAs targeting *RBBP6* or (shNT). Statistical significance was assessed using an ordinary one-way ANOVA with Dunnett’s multiple comparisons test, ****P < 0.0001, *n* = 3. **e** The mRNA level of *MYC* was measured in GSC468 (left) and GSC3565 (right) cells transduced with two separate sgRNAs targeting *RBBP6* or sgNT. Statistical significance was assessed using an ordinary one-way ANOVA with Dunnett’s multiple comparisons test, ****P < 0.0001, *n* = 3. **f** The protein level of MYC was measured in GSC468 and GSC3565 cells transduced with two separate shRNAs targeting *RBBP6* or shNT. **g** The protein level of MYC was measured in GSC468 and GSC3565 cells transduced with two separate sgRNAs targeting *RBBP6* or sgNT. **h** Correlation between *RBBP6* and *MYC* expression in glioma tissues from the TCGA database. **i** Correlation between *RBBP6* and *MYC* expression in glioma tissues from the CGGA database.

To elucidate the molecular mechanisms of RBBP6 in GSCs, we performed RNA-seq following knockdown of *RBBP6* mRNA expression to explore the signaling pathways modulated by RBBP6 (Fig. 3a). Hallmark enrichment analysis revealed that RBBP6 knockdown downregulated several signaling pathways essential for cancer progression, including MYC, TNFA-NFKB, and estrogen response signaling pathways (Fig. 3b). Gene set enrichment analysis (GSEA) showed that genes downregulated in RBBP6 knockdown were highly enriched in the terms MYC targets and translation elongation, while genes upregulated were enriched in apoptotic process (Fig. 3c). Both hallmark enrichment analysis and GSEA demonstrated that RBBP6 modulated the MYC signaling pathway. We then performed qPCR and western blot analyses to confirm that RBBP6 regulated MYC expression (Fig. 3d–g). Analysis of TCGA and Chinese Glioma Genome Atlas (CGGA) datasets revealed that *RBBP6* mRNA levels were positively correlated with *MYC* mRNA levels (Fig. 3h, i). Collectively, these data demonstrate that RBBP6 controls the expression of MYC.

### RBBP6 controlled MYC expression by regulating alternative polyadenylation (APA) events

**Fig. 4:**
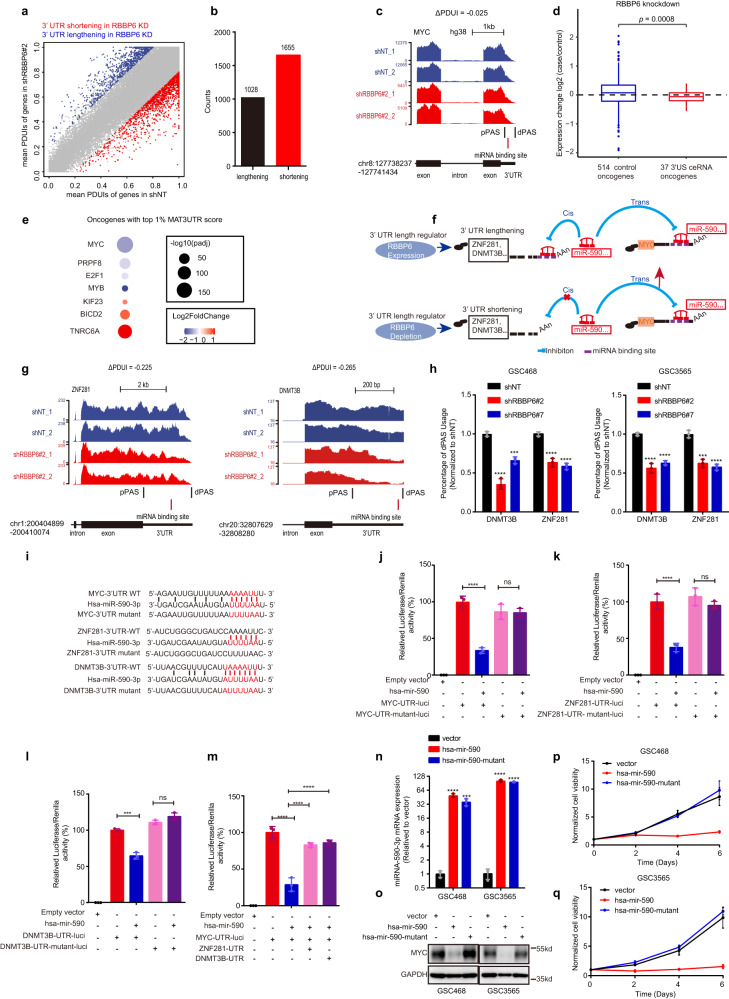
RBBP6 depletion led to APA and decreased MYC expression in *trans*. **a** Scatterplot of PDUIs in GSC468 cells transduced with a shRNA targeting *RBBP6* (shRBBP6#2) or shNT. The red dots indicate genes with significant 3’US and the blue dots indicate genes with 3’UTR lengthening following RBBP6 knockdown. **b** Graph showing the counts of genes with 3’UTR lengthening and shortening. **c** Representative RNA-seq density plots along with ΔPDUI values for MYC. The numbers on the *y*-axis indicate the RNA-seq read coverage. **d** The relative expression levels of OGs that are ceRNAs with 3’US (*n* = 37, right box) are lower than those of control OGs (*n* = 514, left box). Welch’s *t*-test, which accounts for different variances in the two groups being compared, was employed to compare means. To verify the normality assumption for the *t*-test, a Shapiro–Wilk normality test for small sample sizes (*n* < 50) was conducted. **e** Bubble plot showing OGs with MAR3UTR scores in the top 1%. **f** RBBP6 knockdown induces 3’US of a set of genes, allowing miRNAs to repress MYC expression. **g** Representative RNA-seq density plots of *ZNF281* and *DNMT3B*, whose 3’UTRs are shortened in response to RBBP6 knockdown. RNA-seq read coverage is plotted on the *y*-axis. **h** qPCR results of dPAS usage for APA analysis in GSC468 and GSC3565 cells transduced with two separate shRNAs targeting *RBBP6* or a shNT. Statistical significance was assessed using an ordinary one-way ANOVA with Dunnett’s multiple comparisons test, ****P < 0.0001, *n* = 3. **i** Diagram showing miR-590-3p binding sites in the 3’UTRs of *MYC*, *ZNF281*, and *DNMT3B*. **j** Luciferase reporter assay showing miR-590-3p binding to the *MYC* 3’UTR. Renilla luciferase activity was used for luciferase activity normalization. Statistical significance was assessed using an ordinary one-way ANOVA with Dunnett’s multiple comparisons test, ****P < 0.0001, *n* = 3. **k** The luciferase reporter assay showed miR-590-3p binding to the *ZNF281* 3’UTR. Renilla luciferase activity was used for luciferase activity normalization. Statistical significance was assessed using an ordinary one-way ANOVA with Dunnett’s multiple comparisons test, ****P < 0.0001, *n* = 3. **l** The luciferase reporter assay showed miR-590-3p binding to the *DNMT3B* 3’UTR. Renilla luciferase activity was used for luciferase activity normalization. Statistical significance was assessed using an ordinary one-way ANOVA with Dunnett’s multiple comparisons test, ***P < 0.001, *n* = 3. **m** The luciferase reporter assay showed ZNF281, DNMT3B, and MYC competitively binding to miR-590-3p. Statistical significance was assessed using an ordinary one-way ANOVA with Dunnett’s multiple comparisons test, ****P < 0.0001, *n* = 3. **n** Level of miR-590-3p or miR-590-3p mutant in GSC468 and GSC3565 cells transduced with vector, hsa-mir-590, and hsa-mir-590 mutant. Statistical significance was assessed using an ordinary one-way ANOVA with Dunnett’s multiple comparisons test, ***P < 0.001; ****P < 0.0001, *n* = 3. **o** Western blot of MYC in GSC468 and GSC3565 cells transduced with empty vector, hsa-mir-590, or hsa-mir-590 mutant. **p** Cell viability in the GSC468 cells transduced with empty vector, hsa-mir-590, or hsa-mir-590 mutant. Three technical replicates were used for each group. The error bars show the SDs. **q** Cell viability in the GSC3565 cells transduced with empty vector, hsa-mir-590, or hsa-mir-590 mutant. Three technical replicates were used for each group. The error bars show the SDs.

Next, we explored how RBBP6 regulates MYC expression. We considered molecular interactions with other ubiquitin-related targets. MAGE-A11-HUWE1 mediates the polyubiquitination and degradation of PCF11 to regulate APA in cancer, which supports correlation between the ubiquitin‒proteasome pathway and APA^14^. Almost 70% of genes contain multiple polyA sites, resulting in varying mRNA isoforms. The 3’ end processing complex mediates the usage of different polyA sites^15^. APA is an essential posttranscriptional regulation mechanism modulating the translation, stability, and cellular localization of mRNAs^15^, including in glioblastoma^16^. However, the role of different APA regulators in GBM is still largely unknown. RBBP6 has been reported to be involved in the regulation of APA, although the mechanism is unclear^17–19^. Thus, we hypothesized that RBBP6 may regulate *MYC* expression by modulating APA events. We used Dynamic analysis of APA from RNA-Seq (DaPars)^16^ to detect the APA events. We found 2683 3’UTR with APA events in RBBP6 knockdown (shRBBP6) compared with controls. Out of 2683 APA events, 61.68% (1655 of 2683) were shortening and 38.32% (1028 of 2683) were lengthening events (Fig. 4a, b). Overall, there was global 3’ UTR shortening and a tendency to use shorter isoforms in shRBBP6 relative to controls (*P* < 2.2 × 10^−16^; Fig. 4b), which suggested that RBBP6 was a potential APA regulator.

We next asked how RBBP6 induced a decreased expression of MYC. As the 3’UTR length could affect mRNA decay^15^, we hypothesized that APA-mediated 3’UTR lengthening in *MYC* could contribute to its decreased expression in *cis*. However, we found that these 3’UTR changed genes are not enriched in either oncogenes (OGs) or tumor suppressor gene pathways (Supplementary Fig. S3a–c). Besides, our results showed that RBBP6 knockdown did not alter *MYC* 3’UTR length (ΔPDUI = –0.025, Fig. 4c). The 3’UTR is also engaged in competing-endogenous RNA (ceRNA) regulation in *trans*, which downregulates ceRNA through miRNA-mediated repression^20^. We hypothesized that knockdown of RBBP6 induced widespread 3’UTR shortening (3’US) of genes in cancer cells, which in turn resulted in releasing their miRNAs to repress their ceRNA partners in *trans*, such as *MYC*. To test this hypothesis, we used model-based analysis of the *trans*-effect of 3’UTR shortening (MAT3UTR) to predict the *trans*-effect of 3’US to their ceRNA partners^20^. We found 37 *trans*-target OGs of 3’US (top 1% MAT3UTR score). Thirty-seven expressed OGs from 3’US ceRNAs were more likely downregulated than 514 control OGs not in ceRNET (*P* = 8 × 10^−4^, Fig. 4d), indicating an association between 3’US and OG repression. Notably, among 37 OGs, MYC ranked at the top (log_2_FoldChange = –1.23 and adjusted *P* = 1.94 × 10^–179^, Fig. 4e) and was predicted to be a ceRNA of five 3’US genes in GSCs (Fig. 4f). 3’US genes would be expected to release miRNA from their 3’UTR, with the released miRNA available to repress MYC expression (Fig. 4f). The RNA-seq density plots and RT-qPCR analysis results confirmed the 3’US of *ZNF281* and *DNMT3B*, which were predicted to be the *MYC* ceRNAs with the highest confidence scores (Fig. 4g, h). Additionally, we employed the 3’ Rapid Amplification of cDNA Ends (3’ RACE) method to validate that RBBP6 knockdown induces the shortening of the 3’ UTRs of *ZNF281* and *DNMT3B*. This observation aligns with our RT-qPCR results and confirms that RBBP6 knockdown leads to the shortening of the 3’ UTRs of *ZNF281* and *DNMT3B* (Supplementary Fig. S4a–c).

To identify the miRNAs released from the shortened 3’UTRs of *MYC* ceRNAs to inhibit MYC expression, we selected miR-590-3p, with the highest predicted score, for validation (Fig. 4i). Next, we confirmed that RBBP6 knockdown did not change the expression level of miR-590-3p (Supplementary Fig. S4d). This suggests that the downregulation of long 3’UTR isoforms of *ZNF281* and *DNMT3B* mRNAs is not due to an increase in miR-590-3p. We then leveraged the 3’UTR luciferase reporter system to further confirm the binding of miR-590-3p to *MYC* and *MYC* ceRNAs (*DNMT3B* and *ZNF281*). miR-590-3p overexpression specifically decreased *MYC-*, *DNMT3B-* and *ZNF281*-3’UTR-driven luciferase activities and miR-590-3p expression without changing the luciferase activities driven by the 3’UTR binding site mutation (Fig. 4j–l). Reciprocally, overexpression of the 3’UTRs of *MYC* ceRNAs (*DNMT3B* and *ZNF281*) reversed the miR-590-3p-mediated reduction in *MYC*-UTR luciferase activity, supporting the hypothesis that MYC and its ceRNAs competitively bind to miR-590-3p (Fig. 4m). Overexpression of miR-590-3p but not the miR-590-3p mutation (mutant miR-590-3p seed region) in GSCs via a lentiviral vector decreased MYC expression and inhibited GSC proliferation (Fig. 4n–q). Furthermore, we utilized a chemically modified single-strand miRNA inhibitor called miR-590-3p antagomir to inhibit miR-590-3p. Our findings indicate that the suppression of miR-590-3p led to an increase in the mRNA levels of *MYC*, *ZNF281*, and *DNMT3B* in both GSC468 and GSC3565 cells (Supplementary Fig. S4e, f). In addition, we conducted a 3’UTR luciferase reporter assay, which revealed that the inhibition of miR-590-3p by miR-590-3p antagomir enhanced the luciferase activity driven by *MYC-*, *DNMT3B-*, and *ZNF281*-3’UTR. In contrast, no changes in luciferase activity were observed when the miR-590-3p binding sites in the 3’UTR were mutated (Supplementary Fig. S4g–i). These findings indicate that miR-590-3p targets *MYC*, *ZNF281*, and *DNMT3B* mRNAs. Additionally, overexpression of the *DNMT3B* and *ZNF281* 3’UTRs but not their respective mutations (mutant miR-590-3p binding sites) partially reversed the RBBP6 knockdown-induced inhibition of cell proliferation (Supplementary Fig. S4j, k). Moreover, we utilized antisense morpholino to obstruct the usage of distal PASs of the ceRNAs to confirm the ceRNA network under physiological conditions. The antisense morpholino targeting *DNMT3B* or *ZNF281* was employed to block the binding of miR-590-3p to *DNMT3B* or *ZNF281*. This blockade of miR-590-3p binding site on *DNMT3B* or *ZNF281* consequently inhibited the expression of MYC (Supplementary Fig. S5a, b) and impaired GSC proliferation (Supplementary Fig. S5c–f). In summary, RBBP6 modulated MYC expression by regulating the *MYC* ceRNA 3’UTR length.

### RBBP6 ubiquitinated cleavage and polyadenylation specificity factor 3 (CPSF3)

**Fig. 5:**
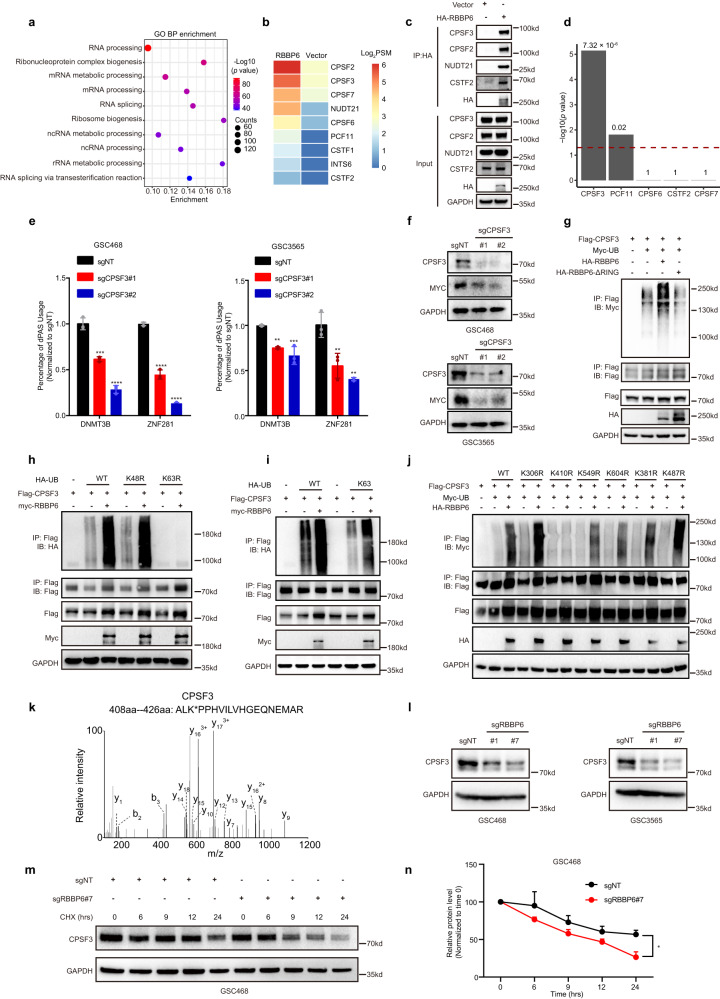
APA interacted with pre-mRNA 3’ end processing factors. **a** GO enrichment analysis of the RBBP6-interacting proteins identified by IP-MS. **b** Heatmap showing that RBBP6 interacted with pre-mRNA 3’ end processing factors identified by IP-MS. **c** HEK293T cells were transfected with HA-RBBP6 plasmids for 48 h and were collected for IP/IB. IB, immunoblotting. **d** The number of the pre-mRNA 3’ end processing factors shortening genes that overlap with *RBBP6* shortening genes follows a hypergeometric distribution. The *y*-axis is the signed Log_10_ *P*-values of the hypergeometric test *P*-values. **e** qPCR results of dPAS usage for APA analysis in GSC468 and GSC3565 cells transduced with two separate sgRNAs targeting *CPSF3* or sgNT. Statistical significance was assessed using an ordinary one-way ANOVA with Dunnett’s multiple comparisons test, **P < 0.01; *** *P* < 0.001; **** *P* < 0.0001, *n* = 3. **f** The protein level of MYC was measured in GSC468 and GSC3565 cells transduced with two separate sgRNAs targeting *CPSF3* or sgNT. **g** HEK293T cells transfected with Flag-CPSF3, Myc-UB, HA-RBBP6 and HA-RBBP6-ΔRING plasmids were collected for IP/IB. **h** HEK293T cells transfected with Flag-CPSF3, Myc-RBBP6, and HA-UB mutant plasmids were collected for IP/IB. **i** HEK293T cells transfected with Flag-CPSF3, Myc-RBBP6, and HA-UB mutant plasmids were collected for IP/IB. **j** HEK293T cells transfected with HA-RBBP6, Myc-UB, and Flag-CPSF3 mutant plasmids were collected for IP/IB. **k** MS spectrum of an identified polyubiquitinated CPSF3 peptide. **l** The protein level of CPSF3 was measured in GSC468 and GSC3565 cells transduced with two separate sgRNAs targeting *RBBP6* or sgNT. **m** The GSC468 cells transduced with a sgRNA targeting *RBBP6* or sgNT followed by CHX (50 µg/mL) treatment for the indicated times were collected for IB. **n** Quantitation of the results shown in **m**. Two-way ANOVA was used for statistical analysis with Sidak’s multiple comparisons test. *P < 0.05, *n* = 3.

To elucidate the molecular mechanisms of RBBP6 in APA event regulation, we performed an unbiased analysis of RBBP6-interacting proteins by immunoprecipitation coupled with mass spectrometry (IP-MS). Gene Ontology (GO) analysis showed that RBBP6-interacting proteins were enriched in RNA process and splicing pathways (Fig. 5a). The IP-MS data indicated that RBBP6 interacted with a set of 3’ UTR processing factors, including CPSF3, CPSF2, NUDT21, and CSTF2 (Fig. 5b; Supplementary Table S2). We confirmed these interactions by co-immunoprecipitation (Co-IP) (Fig. 5c). In addition, we analyzed the public knockdown experiment datasets and also observed significant overlap of 3’UTR changed genes between RBBP6 and CPSF3 (Fig. 5d). Recently, RBBP6 was reported to play a vital role in 3’ end processing, and RBBP6 function is dependent on CPSF3^19^. We found that *CPSF3* KO led to 3’UTR shortening in *MYC* ceRNAs, MYC downregulation, and GSC proliferation defects, consistent with the effects of RBBP6 depletion on GSCs (Fig. 5e, f; Supplementary Fig. S6a, b). Furthermore, *CPSF3* KO induced apoptosis in GSC but not non-neoplastic cells (iPSC-induced astrocyte and NPC), as measured by annexin V staining (Supplementary Fig. S6c–f). Therefore, we focused on the RBBP6-interacting protein CPSF3 for further investigation. RBBP6 is a ubiquitin ligase that interacts with CPSF3, which prompted us to test whether RBBP6 mediates CPSF3 ubiquitination. The overexpression of wild-type RBBP6, but not its enzymatically inactive form RBBP6-ΔRING, led to an increase in polyubiquitination of CPSF3 (Fig. 5g). To characterize the type of CPSF3 ubiquitination mediated by RBBP6, different ubiquitin mutants were co-transfected with RBBP6 and CPSF3 into HEK293T cells followed by CPSF3 immunoprecipitation (IP). Western blot analysis showed that RBBP6 mediated mainly the K63-linked polyubiquitination of CPSF3 (Fig. 5h, i). To characterize the polyubiquitination sites in CPSF3, we overexpressed HA-tagged RBBP6, Flag-tagged CPSF3, and Myc-tagged UB in HEK293T cells, then performed IP with CPSF3, and analyzed the precipitate by MS. The MS results indicated 6 potential ubiquitin-binding sites in the CPSF3 protein (K306, K410, K549, K604, K381, and K487) (Supplementary Table S3). We then generated point mutations at these 6 sites in CPSF3 to further determine the major ubiquitination site. The ubiquitination assay showed that only the K410R mutation abolished RBBP6-mediated CPSF3 polyubiquitination (Fig. 5j, k). *RBBP6* KO decreased CPSF3 protein levels (Fig. 5l). We then tested whether RBBP6 regulates CPSF3 stability through K63-linked polyubiquitination. Overexpression of RBBP6 stabilized CPSF3 (Supplementary Fig. S7a, b), while *RBBP6* KO in GSCs accelerated CPSF3 degradation (Fig. 5m, n).

Next, we conducted CHX experiments to compare half-lives of wild-type and ub-mutant CPSF3 in normal and *RBBP6* KO conditions. Our data showed that RBBP6 KO accelerated the degradation of wild-type CPSF3, but not ub-mutant CPSF3 K410R (Supplementary Fig. S7c–f). Moreover, wild-type CPSF3 exhibited a longer half-life than CPSF3 K410R (Supplementary Fig. S7c–f). To confirm the requirement of RBBP6’s E3 ligase activity for the RBBP6-CPSF3-MYC axis, we conducted a rescue experiment in RBBP6 knockdown GSC468 cells by overexpressing either RBBP6-WT or enzyme activity dead form RBBP6 ΔRING. The overexpression of RBBP6-WT successfully restored the downregulation of CPSF3 and MYC caused by RBBP6 knockdown. In contrast, the overexpression of RBBP6-ΔRING failed to rescue the RBBP6 knockdown-induced downregulation of CPSF3 and MYC (Supplementary Fig. S7g). Taken together, these findings showed that RBBP6 interacted with and stabilized CPSF3 through K63-linked polyubiquitination, and the ubiquitination of CPSF3 by RBBP6 plays a crucial role in CPSF3’s function on MYC.

### Targeting RBBP6 and CPSF3 inhibited GSC growth in vivo

**Fig. 6:**
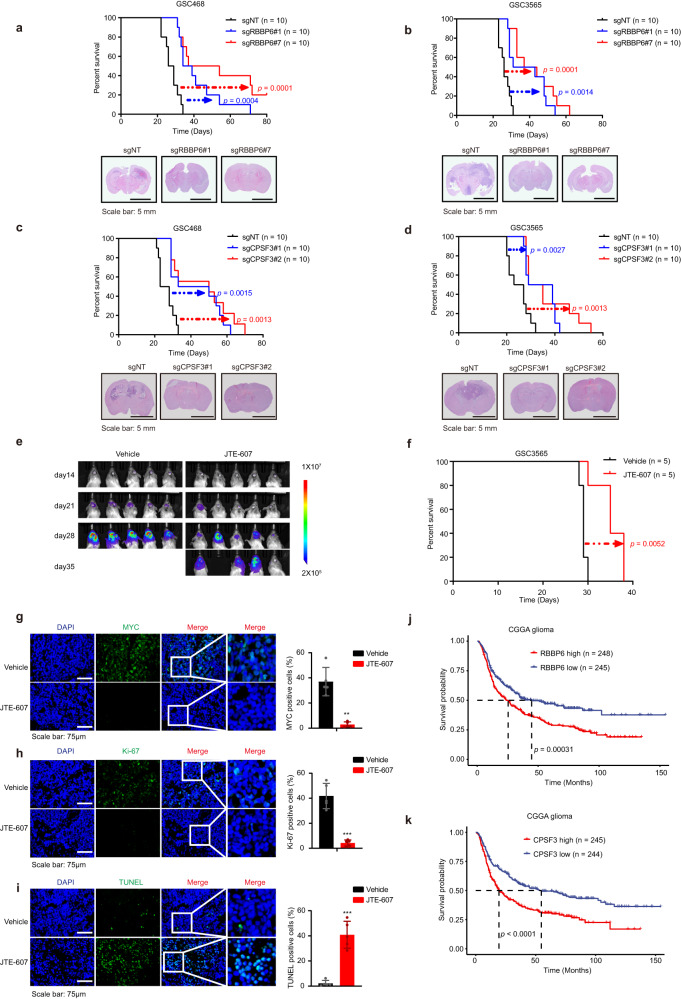
Targeting RBBP6 and CPSF3 inhibitd GSC-derived xenograft growth in vivo. **a** Kaplan‒Meier survival curves of immunocompromised NSG mice bearing intracranial GSC468 cells transduced with sgNT, sgRBBP6#1, or sgRBBP6#7 (Top). Representative images of H&E staining of mouse brains. Brains were isolated after the presentation of the first neurological sign in any cohort (Bottom). **b** Kaplan‒Meier survival curves of immunocompromised NSG mice bearing intracranial GSC3565 cells transduced with sgNT, sgRBBP6#1, or sgRBBP6#7 (Top). Representative images of H&E staining of mouse brains (Bottom). Brains were isolated after the presentation of the first neurological sign in any cohort. **c** Kaplan‒Meier survival curves of immunocompromised NSG mice bearing intracranial GSC468 cells transduced with sgNT, sgCPSF3#1, or sgCPSF3#2 (Top). Representative images of H&E staining of mouse brains (Bottom). Brains were isolated after the presentation of the first neurological sign in any cohort. **d** Kaplan‒Meier survival curves of immunocompromised NSG mice bearing intracranial GSC3565 cells transduced with sgNT, sgCPSF3#1, or sgCPSF3#2 (Top). Representative images of H&E staining of mouse brains (Bottom). Brains were isolated after the presentation of the first neurological sign in any cohort. Scale bars: 5 mm. Log-rank test was used for statistical analysis (**a**–**d**). **e** The bioluminescence image of the vehicle or JTE-607 treated mice. **f** Kaplan‒Meier curve showing survival of mice following implantation with GSC3565 and treatment with vehicle or JTE-607. Log-rank test was used for statistical analysis. **g** Immunofluorescent staining of MYC in the vehicle or JTE-607 treated tumors (left). Quantification of MYC positive cells (right, *t*-test, **P < 0.01, *n* = 4). **h** Immunofluorescent staining of Ki-67 in vehicle or JTE-607 treated tumors (left). Quantification of Ki-67 positive cells (right *t*-test, ***P < 0.001, *n* = 4). **i** Immunofluorescent staining of TUNEL in vehicle or JTE-607 treated tumors (left). Quantification of TUNEL positive cells (right, *t*-test, ***P < 0.001, *n* = 4). Scale bars: 75 μm (**g**–**i**). **j** Kaplan‒Meier curves showing survival based on *RBBP6* mRNA expression in glioma patients from the CGGA dataset. Log-rank test was used for statistical analysis. **k** Kaplan‒Meier curves showing survival based on *CPSF3* mRNA expression in glioma patients from the CGGA dataset. Log-rank test was used for statistical analysis.

To explore the potential benefit of therapeutic targeting of RBBP6 in vivo, we intracranially implanted GSCs bearing *RBBP6* KO sgRNAs or controlled nontargeting sgRNAs into immunocompromised NSG mice. Consistent with the in vivo screening results, *RBBP6* KO inhibited tumor growth and prolonged survival of tumor-bearing mice (Fig. 6a, b; Supplementary Fig. S8a). As CPSF3 appears to be a critical downstream molecule of RBBP6 in GSCs, we investigated the function of CPSF3 in tumor growth in vivo. Concordantly, mice bearing *CPSF3*-KO GSCs displayed longer survival times and smaller tumors than mice bearing control GSCs (Fig. 6c, d).

Due to the lack of a specific RBBP6 inhibitor, we treated GSCs with the CPSF3 enzyme activity inhibitor JTE-607. Treatment with JTE-607 impaired GSC proliferation in a concentration-dependent manner (Supplementary Fig. S8b). To overcome the challenges of poor blood–brain barrier penetration and the short half-life of JTE-607 in vivo (t_1/2_ α = 0.1 h in humans)^21^, we utilized osmotic minipumps to deliver JTE-607 directly into intracranial tumors. This approach allowed us to investigate the anti-tumor efficacy of JTE-607. GSC3565 cells were intracranially transplanted for one week to allow tumor seeding, and then osmotic minipumps were transplanted at the site of injection to deliver JTE-607 locally. JTE-607 administration did not result in a decrease in mouse body weight, indicating an absence of significant toxicity (Supplementary Fig. S8c). JTE-607 treatment suppressed tumor growth and prolonged mouse survival (Fig. 6e, f). Additionally, JTE-607 administration resulted in reduced expression of MYC and Ki-67 in the tumors (Fig. 6g, h). Moreover, treatment with JTE-607 demonstrated its ability to induce tumor cell apoptosis, as measured by the TUNEL assay (Fig. 6i). We then performed in silico analysis to confirm that high RBBP6 and CPSF3 expression correlated with worse survival in glioma patients (Fig. 6j, k). Collectively, these results demonstrated that RBBP6 and CPSF3 are potential therapeutic targets in glioblastoma.

## Discussion

High-throughput screening of established cell lines in vitro to discover putative genes that regulate cell proliferation or death is a traditional approach to cancer drug discovery. To date, this approach has led to the successful development of many antitumor drugs, but the overall success rates of these drugs remain low. One of the major reasons is the vast differences between in vitro culture conditions and the in vivo tumor growth microenvironment. To overcome this limitation, we performed parallel in vivo and in vitro CRISPR/Cas9 functional screens to investigate targetable dependencies of GSCs and identified RBBP6 as a consistent regulator of GSC maintenance and tumor formation in vitro and in vivo.

The APA mechanism plays a crucial role in posttranscriptional regulation by modulating mRNA stability, translation, and cellular localization^15^. The majority of mRNAs are cleaved and polyadenylated in their 3’UTRs, events that can result in the production of different mRNA isoforms with varying 3’UTR lengths to evade or be affected by miRNA-mediated regulation. The ratio of proximal polyA sites to dPASs varies across tissues and diseases^22^. APA events are regulated by four complexes: CPSF, cleavage factors I and II (CF I and II), and cleavage stimulation factor (CSTF)^23^. CPSF3 constitutes a central element of the seven-subunit CPSF complex, playing a crucial role in the cleavage and polyadenylation processes of mRNAs. The assembly of CPSF3 with specific proteins forms the processing machinery required to activate its endonuclease activity. Depletion of CPSF3 results in significant alterations in APA and gene expression. Recent studies have suggested that CPSF3 could serve as a prognostic marker and potential therapeutic target in various cancers, including non-small cell lung cancer^24^, triple-negative breast cancer^25^, prostate cancer^26^, colorectal cancer^27^, acute myeloid leukemia, and Ewing’s Sarcoma^28^. Ross et al. utilized phenotypic screening in conjunction with chemical genetics to identify CPSF3 as the target of JTE-607. JTE-607-mediated inhibition of CPSF3 led to alterations in the expression of known downstream effectors in both acute myeloid leukemia and Ewing’s Sarcoma. Additionally, treatment with JTE-607 induced apoptosis and impeded tumor growth in mouse xenograft models^28^. Consequently, the CPSF3-mediated APA processing pathway holds promise as a target for cancer treatment.

Dysregulation of APA contributes to cancer initiation and progression. It was reported that the knockdown of one of the CPSF complex members, NUDT21 (also called CPSF5), led to 3’US of oncogenes and increased glioblastoma cell proliferation^16^. Here, we found that RBBP6 regulates APA by ubiquitinating CPSF3. Depletion of RBBP6 or CPSF3 resulted in 3’US of the ceRNA of the OG MYC, thereby reducing MYC expression and leading to inhibition of tumor growth. Our finding indicated that the ubiquitination of CPSF3 by RBBP6 plays a crucial role in CPSF3’s function on MYC. Two recent papers^19,29^ showed that RBBP6 interacts with CPSF3 and promotes its endonuclease activity, independently of its RING domain. In comparison with the substantial quantity of CPSF3 protein utilized in the in vitro assays, the endogenous protein levels of CPSF3 within cells are significantly lower. Consequently, protein stability may play a more critical role in CPSF3’s functionality in cellular contexts. These results indicated that different APA regulators may play distinct roles in glioblastoma development and progression. Our identification of the dependency on the RBBP6/CPSF3-APA-MYC axis in glioblastoma offers novel strategies for lethal cancers.

RBBP6 was originally identified as an Rb-interacting protein and later proven to be a ubiquitin E3 ligase. Recent studies have indicated that RBBP6 may be a prognostic marker for and potential therapeutic target in many cancers, including cervical carcinoma^30^, colorectal cancer^31^ and non-small cell lung cancer^32^. Few RBBP6 ubiquitination substrate proteins have been reported thus far, and these include Zinc Finger And BTB Domain Containing 38 (ZBTB38)^33^, NFKB Inhibitor Alpha (NFKBIA)^31^, Y-Box Binding Protein 1 (YBX1)^34^ and Growth Factor Receptor Bound Protein 2 (GRB2)^35^. We demonstrated here that CPSF3 is a novel ubiquitination substrate of RBBP6. RBBP6 mediates K63-linked polyubiquitination of CPSF3 and promotes its stability. K48- and K63-linked chains are the two most abundant ubiquitin chain types. Unlike K48-linked ubiquitination, which is a typical signal for proteasomal degradation, K63-linked ubiquitination has many well-studied nondegradative roles. For example, K63-linked ubiquitination of IKK enhances its binding to the activation upstream kinase TAK1^36^, and K63-linked ubiquitination of Akt promotes its cell membrane translocation^37^. Interestingly, a recent study also reported that the E3 ligase Pellino-1 stabilized the transcription factors Slug and Snail via K63-linked ubiquitination^38^, which is consistent with our findings, although the exact mechanism requires further investigation.

**Fig. 7:**
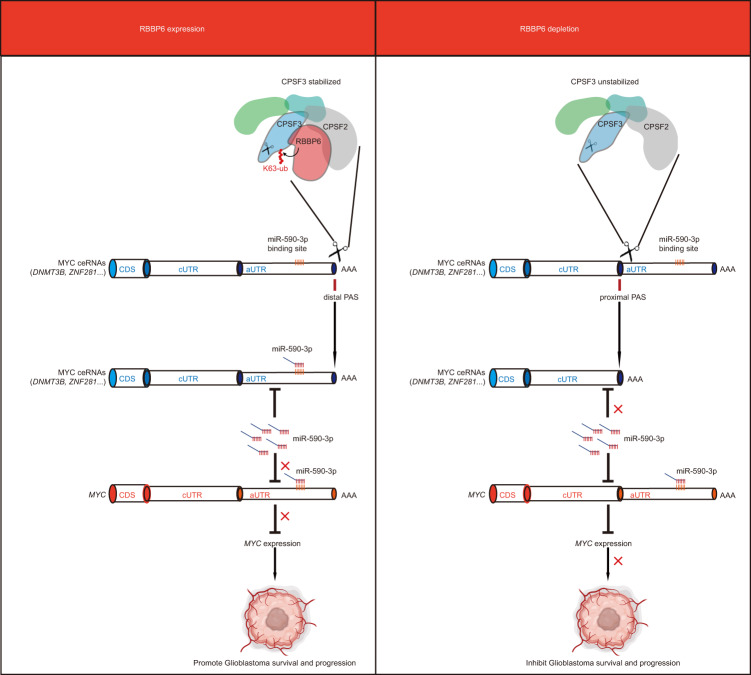
Working model of RBBP6 in regulating GSCs. RBBP6 regulates the maintenance of GSCs. Depletion of RBBP6 leads to the elimination of K63-linked ubiquitination of CPSF3, resulting in the destabilization of CPSF3 and the subsequent shortening of the 3’ UTRs of *MYC* ceRNAs. This shortening promotes the release of miR-590-3p from the truncated UTRs, thereby reducing MYC expression and hindering the survival and progression of GSCs.

In summary, by utilizing an in vitro and in vivo CRISPR screening, we identified the dependency on the RBBP6/CPSF3-APA-MYC axis in glioblastoma (Fig. 7). Our data suggest that approaches to modulate APA in GSCs by targeting RBBP6 or CPSF3 showed high therapeutic efficacy, offering novel strategies for glioblastoma treatment.

## Materials and methods

### GSC derivation

The GSC468, GSC738, and GSC3565 cell lines were generated in our laboratory as reported previously^39^. Patient-derived xenografts were generated and maintained as a reproducible source of GSC cells. The glioblastoma primary cells GBM12388157, and GBM12479390 in this study were obtained from the Department of Neurosurgery, the Second Affiliated Hospital of Zhejiang University School of Medicine. All glioblastoma tissues were obtained from excess surgical resection samples from patients at the Second Affiliated Hospital Zhejiang University School of Medicine with appropriate consent and in accordance with an IRB-approved protocol (IR2022453). All GSC and primary GBM cells were cultured as neurospheres in neurobasal medium (Gibco, Cat# 12349-015) supplemented with 2% B27 supplement (Gibco, Cat# 12587-010), 1% GlutaMax Supplement (Gibco, Cat# 35050-061), 1% sodium pyruvate (Gibco, Cat# 11360-070), 1% penicillin/streptomycin (Invitrogen, Cat# SV30010), 20 ng/mL basic human fibroblast growth factor (R&D systems, Cat# 4114-TC), and 20 ng/mL human epidermal growth factor (R&D systems, Cat# 236-EG). Short Tandem Repeat (STR) analyses were performed to authenticate the identity of each cell line used in this article. Mycoplasma testing was performed by qPCR with cellular supernatants on a yearly basis. Cells were grown for fewer than 20 in vitro passages from xenografts.

### Other cell models

The HEK293T cells were purchased from the American Type Culture Collection (ATCC, Cat# CRL-3216). HEK293T cells were cultured in DMEM (Gibco, Cat# C11995500CP) supplemented with 1% GlutaMax Supplement, 1% penicillin/streptomycin and 10% fetal bovine serum (CellMax, Cat# SA211.02). The iPSC cells were purchased from Cellapy (Cellapy, Cat# CA4002106). iPSC-NPCs were differentiated from the iPSC cells using serum-free STEMdiff™ SMADi Neural Induction Kit (StemCell Technologies, Cat# 08581) according to the manufacturer’s instructions. Astrocytic precursors were generated from iPSC-NPCs using STEMdiff™ Astrocyte Differentiation Kit (StemCell Technologies, Cat# 100-0013) according to the manufacturer’s instructions. These astrocytic precursors are then matured further into astrocytes using STEMdiff™ Astrocyte Maturation Kit (StemCell Technologies, Catalog# 100-0016) according to the manufacturer’s instructions. iPSC-astrocytes were cultured in DMEM supplemented with 1% GlutaMax Supplement, 1% penicillin/streptomycin, and 10% fetal bovine serum. iPSC-NPC were cultured in a neurobasal medium supplemented with 2% B27 supplement, 1% GlutaMax Supplement, 1% sodium pyruvate, 1% penicillin/streptomycin, 20 ng/mL basic human fibroblast growth factor, and 20 ng/mL human epidermal growth factor. STR analyses were performed to authenticate the identity of each cell line used in this article. Mycoplasma testing was performed by qPCR with cellular supernatants yearly.

### Animal experiments

All mouse experiments were performed under an animal protocol approved by the Institutional Animal Care and Use Committee of Westlake University and in accordance with the relevant guidelines. Intracranial transplantation of GSCs was performed as previously described^39^. In brief, GSC spheres were dissociated into single cells, and 10,000 cells were intracranially injected into the right cerebral cortex of individual NSG immunocompromised mice. When any mouse was observed to exhibit neurological signs or signs of morbidity, including lethargy, gait changes, hunched posture, and weight loss, we sacrificed all mice in that cohort. To compare tumor growth in vivo, we isolated brains from mice transplanted with GSCs on the day that neurological signs or signs of morbidity were observed. Then, the brains were used for histologic analysis by H&E staining. In parallel survival experiments, mice were observed until the development of neurological signs or signs of morbidity.

### Evaluation of JTE-607 in GBM model

GSC spheres were dissociated into single cells, and 10,000 cells were intracranially injected into the right cerebral cortex of individual NSG immunocompromised mice. Osmotic minipumps (ALZET, Cat# 21-3032) were implanted for the direct delivery of the vehicle or JTE-607 (dissolved in water, 7.143 μg per day per mouse) to the tumor site through a brain infusion kit (ALZET, Cat# 0008663) on one week after intracranial GSC injection.

### Immunofluorescence

The tissues were fixed overnight at 4 °C using 4% paraformaldehyde, followed by a 48-h incubation in 30% sucrose. Then, the tissues were embedded in an OCT embedding medium. Cryosections with a thickness of 10 µm were prepared for the immunofluorescence experiment. For immunofluorescence staining, the sections were fixed with 4% paraformaldehyde at room temperature for 10 min. Subsequently, the sections were washed three times with 1× PBS. To permeabilize the sections, 0.25% Triton-X100 was applied at room temperature, followed by another three washes with 1× PBS. After the washing steps, the sections were blocked with 5% serum derived from the donkey and then washed with 1× PBS. Staining for MYC (Cell signaling Technology, Cat# D3N8F, 1:200) or Ki-67 (Proteintech, Cat# 27309-1-AP, 1:200) was carried out at room temperature for 1 h. Following the staining, the sections were washed three times and cover-slipped using a fluorescent mounting medium containing DAPI (ZSGB-BIO, Cat# ZLI-9557). To detect apoptosis in the tumors, the sections were processed using a TUNEL kit (Beyotime, Cat# C1086) according to the manufacturer’s instructions.

### Chemicals and oligos

JTE-607 was purchased from MedChemExpress (Cat# HY-110133). The antisense morpholino was purchased from Gene Tools. The antisense oligonucleotide sequences employed in this study are provided below: NC-morpholino: 5’-CCTCTTACCTCAGTTACAATTTATA-3’; ZNF281- morpholino: 5’-AATTTTGGATCAGCCCAGATGGAGA-3’; DNMT3B- morpholino: 5’-GGCTCCAGTTACAAAAAAAATTTTA-3’. Hsa-miR-590-3p antagomir was purchased from Ribobio (Cat# miR30004801-4-5).

### CRISPR/Cas9 library

The CRISPR/Cas9 library in this study included 100 nontargeting controls and 3704 unique sgRNAs individually targeting 463 ubiquitin E3 ligases and deubiquitinases. A customized single-strand sgRNA oligonucleotide pool was synthesized by Twist Bioscience, amplified by PCR and then inserted into the lentiCRISPR-v2 vector (lentiCRISPR-v2, RRID:Addgene_52961, Addegene) using the NEBuilder HiFi DNA Assembly Cloning Kit (NEB, Cat# E5520). Next-generation sequencing was performed to ensure sgRNA abundance.

### Lentivirus production

HEK293T cells were cotransfected with a lentiviral expression vector, the packaging plasmid psPAX2 (psPAX2, RRID:Addgene_12260, Addegene) and the envelope plasmid pMD2.G (pMD2.G, RRID:Addgene_12259, Addegene) using polyethylenimine (PEI) transfection reagent (Polyscience, Cat # 23966-1) following the manufacturer’s instructions. Lentiviral particles were collected 48 h after the medium change and concentrated using the lentivirus concentration kit (Genomeditech, Cat# GM-040801-100).

### CRISPR screen of GSCs

GSC spheres were dissociated into single cells with TrypLE (ThermoFisher Scientific, Cat# 12604021) and were transduced with the lentiviral CRISPR library at a multiplicity of infection of 0.3–0.5 before puromycin selection. For the in vitro screens, GSCs were cultured for 1–2 weeks to ensure at least 10-fold cell doubling. For the in vivo screens, GSCs were implanted into the brains of NSG mice (1 million GSCs per mouse), and the brains were harvested when the mice showed neurological signs. Genomic DNA was isolated from GSCs at the experimental start points as well as from GSCs harvested at the experimental endpoints to prepare the library for next-generation sequencing.

### Analysis of CRISPR screen data

FASTQ files were trimmed with Trim Galore (Trim Galore, RRID:SCR_011847). The trimmed FASTQ files were then analyzed using MAGeCK software to obtain the read count of each sgRNA. MAGeCK robust rank aggregation (RRA) algorithm was used to identify significantly enriched and depleted sgRNAs and genes by comparing the in vitro cultured samples and in vivo tumor samples with the control samples.

### Plasmids

The following sgRNA sequences were chosen from our CRISPR library or designed with CHOP-CHOP (CHOPCHOP, RRID:SCR_015723). SgRNA sequences were inserted into the lentiCRISPR v2 plasmid (lentiCRISPR v2, RRID:Addgene_52961, Addgene). SgRNAs used in this study were sgNT: 5’-CTCTGCTGCGGAAGGATTCG-3’, sgRBBP6#1: 5’-AAGTCGAACTGAACCAGCGA-3’, sgRBBP6#7: 5’-AAGTCGAACTGAACCAGCGA-3’, sgCPSF3#1: 5’-ATGTTCATGATTGAGATCGC-3’, sgCPSF3#2: 5’-ATTCATAGACTAACCACATG-3’. The shRNA sequences were inserted into the pLKO.TRC.1 plasmid (pLKO.TRC.1, RRID: Addgene_10878, Addgene). ShRNAs used in this study were shNT: 5’-CAACAAGATGAAGAGCACCAA-3’, shRBBP6#2: 5’- GACTCTCCTTCTCGGAATAAA-3’, shRBBP6#7: 5’-GATGACTCTTCCGCGTCTATT-3’, shRBBP6#UTR: 5’-GGGTCTCTGGATTATTGTT-3’.

### Quantitative RT-PCR

Total cellular RNA was extracted with TRIzol reagent (Invitrogen, Cat# 15596018) according to the manufacturer’s instructions. Then, RNA was reverse transcribed to cDNA with a cDNA reverse transcription kit (Novoprotein, Cat# E047-01B). Additionally, miRNA was reverse transcribed to cDNA with a miRNA 1st Strand cDNA Synthesis Kit (Vazyme, Cat# MR101-01) and specific probes (miR-590-3p: GTCGTATCCAGTGCA-GGGTCCGAGGTATTCGCACTGGATACGACACTAGC, *GADPH*: GGCTGTTGTCAT-ACTTCTCATGG; *RNU6*: AGGGGCCATGCTAATCTTCT). qPCR was performed with Novostart SYBR qPCR Supermix Plus (Novoprotein, Cat# E096) in a Bio-Rad CFX instrument. The qPCR primers used in this study were as follows: *GAPDH* forward primer: 5’-GGAGCGAGATCCCTCCAAAAT-3’, *GAPDH* reverse primer: 5’-GGCTGTTGTCATACTTCTCATGG-3’; *18* *S* forward primer: 5’-GGCCCTGTAATTGGAATGAGTC-3’; *18* *S* reverse primer: 5’-CCAAGATCCAACTACGAGCTT-3’; *RBBP6* forward primer: 5’-CATCTCCCTCTGCGACTTAAAG-3’; *RBBP6* reverse primer: 5’-TAGTTGCCATCGCTGGTTCAG-3’; *MYC* forward primer: 5’-GGCTCCTGGCAAAAGGTCA-3’; *MYC* reverse primer: 5’- CTGCGTAGTTGTGCTGATGT-3’; *DNMT3B* (coding sequence, CDS) forward primer: 5’-AGGGAAGACTCGATCCTCGTC-3’; *DNMT3B* (CDS) reverse primer: 5’-GTGTGTAGCTTAGCAGACTGG-3’; *DNMT3B* (UTR) forward primer-: 5’-TGGAGCCACGACGTAACAAA-3’; *DNMT3B* (UTR) reverse primer: 5’-GCATCCGTCATCTTTCAGCC-3’; *ZNF281* (CDS) forward primer: 5’-AGGACCTCAGTATTCTCCACC-3’; *ZNF281* (CDS) reverse primer: 5’- CCATCTCCAACCAAAGAAGGTTT-3’; *ZNF281* (UTR) forward primer: 5’- TGCTTTACTCTCAGGAAAGTGT-3’; *ZNF281* (UTR) reverse primer: 5’-TGTTACAGTTGAGATCAAGAGAGGG-3’; miR-590-3p forward primer: 5’-GCGCGCGCGCTAATTTTATGTATAA-3’; miR-590-3p reverse primer: 5’-CAGTGCAGGGTCCGAGGTAT-3’.

APA analysis of a single gene was performed as previously described^16^. In brief, the levels of transcripts amplified with common primers targeting the CDS were used for normalization to the total transcript level. The distal primers targeted sequences just upstream of the dPAS and were used to detect long transcripts that used the dPAS. Values were calculated as previously described^16^. ΔCT (common or distal) = CT_common or distal_−CT_GAPDH_. ΔΔCT = ΔCT_distal_− ΔCT_common_. Normalized ΔΔΔCT = ΔΔCT_average of case_ – ΔΔCT_average of control._

### Apoptosis assay

The apoptosis assay was performed with Annexin V-Alexa Fluor 647 (Yeasen, Cat# 40304ES60). Samples were analyzed using a Beckman Coulter Cytoflex Cytometer.

### ELDA

The neurosphere formation capacity was assayed by ELDA. In brief, different numbers of cells per well (100, 50, 20, 10, and 5) were plated into 96-well plates. Seven days later, the number of each well containing neurospheres was recorded. ELDA software was used to analyze the number of wells containing neurospheres as previously described^40^.

### Cell proliferation assay

Twenty-five hundred cells per well were plated in 96-well plates. Cell viability was measured with a CellTiter-Glo Luminescent Cell Viability Assay Kit (Promega, Cat# G7572) at the indicated times.

### Western blotting analysis

Cells were collected, washed, lysed with RIPA buffer (Beyotime, Cat# P0013C) and incubated on ice for 30 min. Lysates were centrifuged at 4 °C for 15 min at 12,000 rpm, and the supernatants were collected. A Bradford (Beyotime, Cat# P0006C) kit was used to determine protein concentrations. Samples containing equal amounts of protein were mixed with LDS Sample Buffer (Invitrogen, Cat# B0007), boiled for 10 min, separated using SDS-PAGE, and then transferred onto PVDF membranes. The membranes were blocked with 5% nonfat milk for 1 h. Next, the membranes were incubated with a primary antibody at 4 °C overnight. The membranes were washed with TBST buffer and were then incubated with secondary antibodies in 5% nonfat milk for 1 h. For all western blot analyses, membranes were imaged using Bio-Rad Image Lab software (Image Lab, RRID:SCR_003073). The antibodies used in this study wereRBBP6 (Bethyl Laboratories, Cat# A304-975A), CPSF3 (Proteintech, Cat# 11609-1-AP), CPSF2 (Proteintech, Cat# 17739-1-AP), NUDT21 (Proteintech, Cat# 10322-1-AP), CSTF2 (Proteintech, Cat# 26825-1-AP), HA-tag (Cell signaling Technology, Cat# 3724 S), Flag-tag (Sigma, Cat # F1804), MYC-tag (Proteintech, Cat # 16286-1-AP), MYC (Cell signaling Technology, Cat# D3N8F), GAPDH (Proteintech, Cat# 60004-1-Ig).

### IP experiments

Cells were collected, washed, lysed with NP-40 lysis buffer (Beyotime, Cat# P0013F) and incubated on ice for 30 min. Lysates were centrifuged at 4 °C for 15 min at 12,000 rpm, and the supernatants were collected. A Bradford (Beyotime, Cat# P0006C) kit was used to determine protein concentrations. For IP experiments, samples containing one milligram of protein were incubated with the indicated anti-Flag-M2 beads (Sigma, Cat# M8823) or anti-HA beads (ThermoFisher Scientific, Cat# 88838) for 1 h at room temperature. After incubation, the beads were washed with lysis buffer five times. After washing, the beads were mixed with LDS Sample Buffer (Invitrogen, Cat# B0007) and boiled for 10 min for western blot analysis.

### Luciferase reporter assay

The indicated plasmids were transfected into HEK293T cells (ATCC, Cat# CRL-3216) for 48 h. Then, we used a Dual-Luciferase Reporter Gene Assay Kit (Yeasen, Cat# 11402ES60) to measure firefly and Renilla luciferase activity.

### 3’ RACE

The full length 3’UTR mRNA were generated with GoScript™ Reverse Transcriptase (Promega, Cat# A5001) kit, following the manufacturer’s protocol with Oligo dT18-N(22) primer. GSP-1 primers and Oligo N(22) primer were used to perform the first-round PCR. GSP-2 primers and Oligo N(22) primer were used to perform the second-round PCR. The 3’RACE primers used in this study were as follows: Oligo dT18-N(22) primer: 5’-CTGATCTAGAGGTACCGGATCCTTTTTTTTTTTTTTTTTT-3’; Oligo N(22) primer: 5’-CTGATCTAGAGGTACCGGATCC-3’; *ZNF281* GSP-1 primer: 5’-GGAGTGTGGTTTCGGCCAA-3’; *ZNF281* GSP-2 primer: 5’- AGTGTGGTTTCGGCCAATCT-3’; *DNMT3B* GSP-1 primer: 5’- TCTTTGGCTTTCCTGTGCAC-3’; *DNMT3B* GSP-2 primer: 5’- TGGCTTTCCTGTGCACTACA-3’. The PCR products were further sequenced.

### RNA-seq analysis

Total RNA was extracted with an RNeasy Small RNA Isolation Kit with Spin Columns (Beyotime, Cat# R0028) according to the manufacturer’s instructions. RNA samples, namely, 4 samples of GSC468 cell lines under two different conditions (Control: GSC468_shNT; Case: GSC468_RBBP6 knockdown) with 2 biological replicates per condition, were sequenced on the Illumina platform, and data were obtained in FASTQ format. For each FASTQ file, quality checks were conducted using FastQC (FastQC, RRID:SCR_014583)^41^. Contaminating data, such as low-quality reads, adaptor sequences, and poor-quality bases, were removed with Trimmomatic software (Trimmomatic, RRID:SCR_011848)^42^, and 252,765,061 clean reads were generated. The trimmed reads were mapped to the human reference genome (GRCh38) using STAR (STAR, RRID:SCR_004463)^43^ and were then sorted by SAMtools (SAMtools, RRID:SCR_002105)^44^. Subsequently, the uniquely mapped reads were quantified by featureCounts (featureCounts, RRID:SCR_002105)^45^. The transcript per million (TPM) values were calculated using RSEM. The genes with TPM < 1 in more than 80% of samples were removed. The remaining genes were then used to perform downstream analysis. DEG analysis was performed with the DESeq2 package (DESeq, RRID:SCR_000154)^46^.

### APA event analysis

We used DaPars2 to identify the most significant APA events between the shNT and RBBP6 knockdown conditions. To quantify APA events, we first downloaded the human gene annotation file (GRCh38) from UCSC and extracted a 3’UTR annotation for each transcript. Subsequently, .wig files were generated using STAR (STAR, RRID:SCR_004463). The PDUI values were extracted by the DaPars2 algorithm (version 2.0)^47^ with the .wig files. A PDUI score close to 0 indicates that the gene tends to use a proximal PAS, whereas a PDUI score close to 1 indicates that the gene tends to use a dPAS. Statistical significance was assumed when the adjusted *P*-value of the PDUI difference was less than 0.05 and the absolute mean PDUI difference was greater than 0.2.

### *Trans*-effect analysis of 3’US

MAT3UTR software^20^ was used to quantify the *trans*-effect of 3’US. First, we extracted the 3’UTR information of transcripts from UCSC. Second, we collected the miRNA binding data from TarBase^48^, miRecords^49^, miRTarBase^50^, and TargetScanHuman version 6.2^51^; these data contained the transcript ID, gene name, miRNA family name, chromosome, start and end coordinate of the binding region, and strand information. Third, we assumed coexpression when a pair of transcripts shared at least five miRNA binding sites in their 3’ UTRs. We identified the potential ceRNA partners of the given genes with 3’US. Finally, we used the script ‘MAT3UTR.py’ to estimate the *trans*-effect of 3’US for each mRNA and miRNA pair. Enrichment analysis was conducted to examine the ceRNA partner genes’ association with OGs. The OGs utilized in this study were determined by the TUSON algorithm, which identified residue-specific activating mutations in over 8200 tumor/normal pairs through genome sequencing. The genes were ranked based on their TUSON prediction *P*-values, with the top 1000 genes (*P* < 0.01) considered as the reference oncogenes for the enrichment analysis. To enhance statistical power, we selected 551 highly expressed OGs (TPM > 1) for further analysis. Among these 551 OGs, 37 were identified as 3’UTR ceRNAs in our study, while 514 were not in the ceRNA networks (ceRNET). Welch’s *t*-test, which accounts for different variances in the two groups being compared, was employed to compare means. To verify the normality assumption for the *t*-test, a Shapiro–Wilk normality test for small sample sizes (*n* < 50) was conducted. All statistical analyses were performed using R (version 3.6).

### APA regulatory analysis

The 3’ end processing factors datasets were retrieved from NCBI GEO (GSE151919 and GSE149204) and ENCODE (ENCSR594DNW vs ENCSR067GHD, ENCSR815JDY vs ENCSR856ZRV, ENCSR895BTE vs ENCSR913CAE). We then used DaPars2 to identify the most significant APA events. We applied a hypergeometric test to test for significant overlap between the pre-mRNA 3’ end processing factors shortening genes and the *RBBP6* shortening genes.

### Supplementary information

Supplementary information

Supplementary tableS1

Supplementary tableS2

Supplementary tableS3

## Data Availability

All the RNA-seq raw sequencing data have been deposited into the National Center for Biotechnology Information Sequence Read Archive under accession number PRJNA914900. The expression data of RBBP6 in both glioblastoma (GBM) and non-tumor samples was obtained from the Gliovis data portal^52^. The data of RBBP6 expression in GSCs and NSCs can be found on the Gene Expression Omnibus (GEO) under the accession number GSE119834. H3K27ac ChIP-seq data can be accessed on the GEO with the accession numbers GSE54792 and GSE119755. For survival and gene correlation analyses, data were obtained from the CGGA and TCGA from the Gliovis data portal.
