# Supplementary material for: RBBP6 maintains glioblastoma stem cells through CPSF3-dependent alternative polyadenylation
Source: Cell Discov. 2024 Mar 19;10:32. doi: 10.1038/s41421-024-00654-3 (PMC10951364; doi:10.1038/s41421-024-00654-3)
Supplement: Supplementary file 1 — Supplementary information [file 41421_2024_654_MOESM1_ESM.pdf]

Supplementary Fig. S1

a

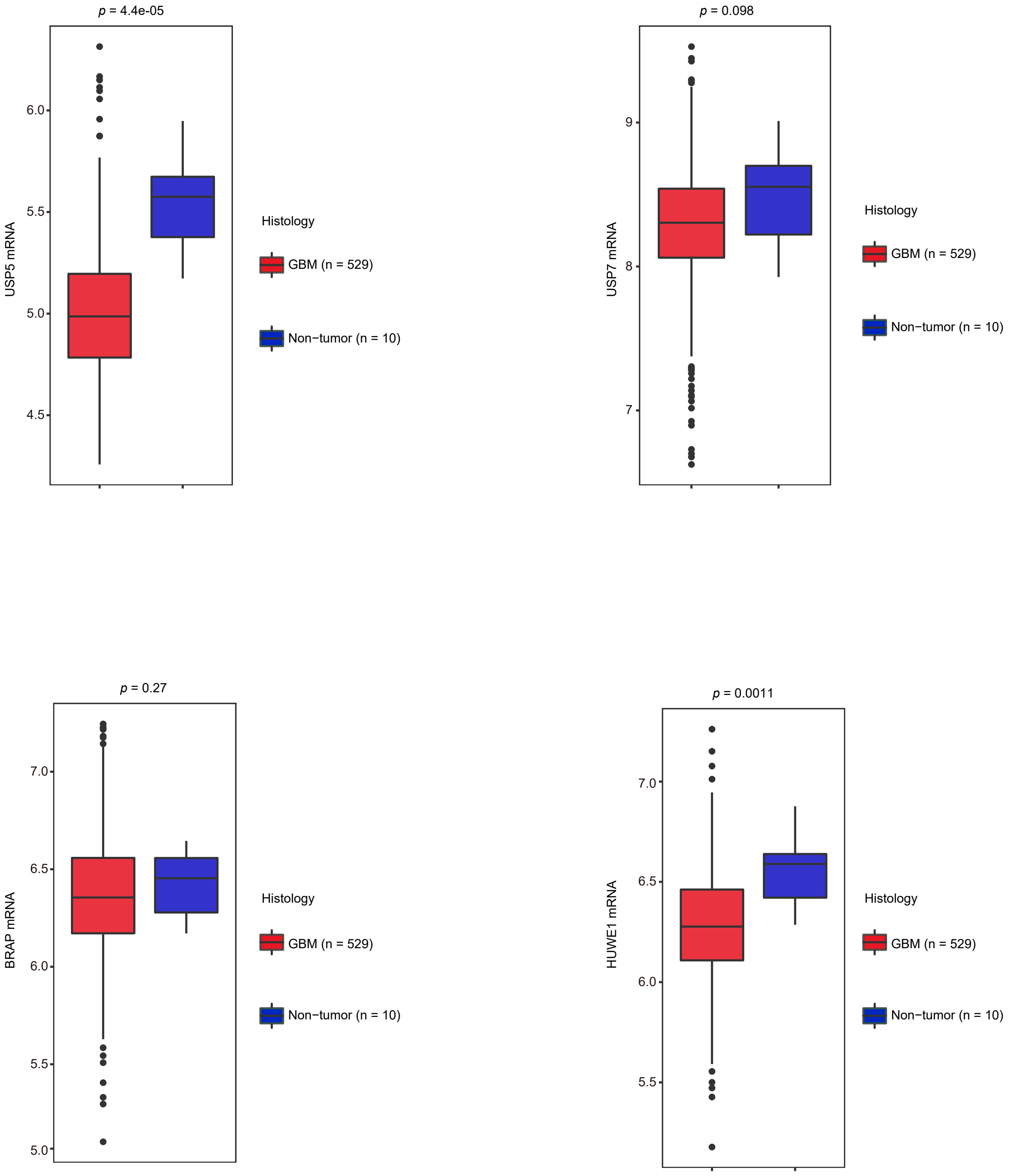

Supplementary Fig. S1 The expression levels of other CRISPR screen hits.

a *USP5*, *USP7*, *BRAP*, and *HUWE1* mRNA expression level in TCGA GBM dataset (U133A). *UHRF1* expression is not detectable in the same dataset. Statistical significance was assessed using a t test.

Supplementary Fig. S2

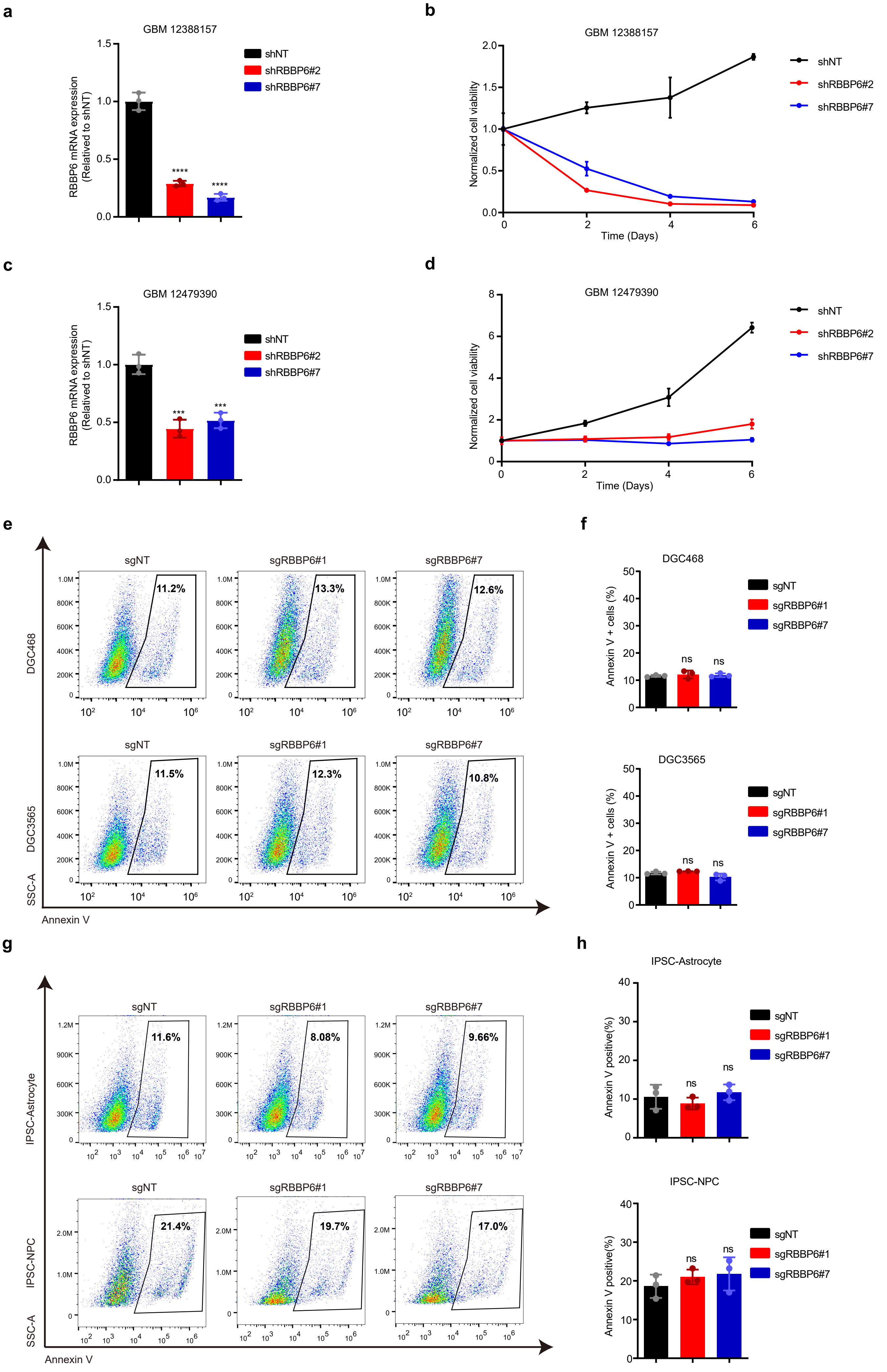

Supplementary Fig. S2 RBBP6 knockdown inhibited GBM cell proliferation.

a The mRNA level of *RBBP6* in GBM12388157 cells transduced with two separate shRNAs targeting *RBBP6* or a non-targeting shRNA (shNT). Statistical significance was assessed using an ordinary one-way ANOVA with Dunnett's multiple comparisons test, **** p <0.0001, n = 3. **b** Cell viability in the GBM12388157 cells transduced with two separate shRNAs targeting *RBBP6* or a non-targeting shRNA (shNT). Three technical replicates were performed for each group. Error bars show SDs. **c** The mRNA level of *RBBP6* in GBM12479390 cells transduced with two separate shRNAs targeting RBBP6 or a non-targeting shRNA (shNT). Statistical significance was assessed using an ordinary one-way ANOVA with Dunnett's multiple comparisons test, *** p <0.001, n = 3. **d** Cell viability in the GBM12479390 cells transduced with two separate shRNAs targeting *RBBP6* or a non-targeting shRNA (shNT). Three technical replicates were performed for each group. Error bars show SDs. **e, f** Annexin V staining of DGC3565 and DGC468 cells transduced with two separate sgRNAs targeting *RBBP6* or a nontargeting sgRNA (sgNT). Quantification of Annexin-V staining using an ordinary one-way ANOVA with Dunnett's multiple comparisons test, ns, no significant; n = 3. **g, h** Annexin V staining of IPSC-Astrocyte and IPSC-NPC cells transduced with two separate sgRNAs targeting *RBBP6* or a nontargeting sgRNA (sgNT). Three technical replicates were used for each condition. Quantification of Annexin-V staining using an ordinary one-way ANOVA with Dunnett's multiple comparisons test, ns, no significant; n = 3.

Supplementary Fig. S3

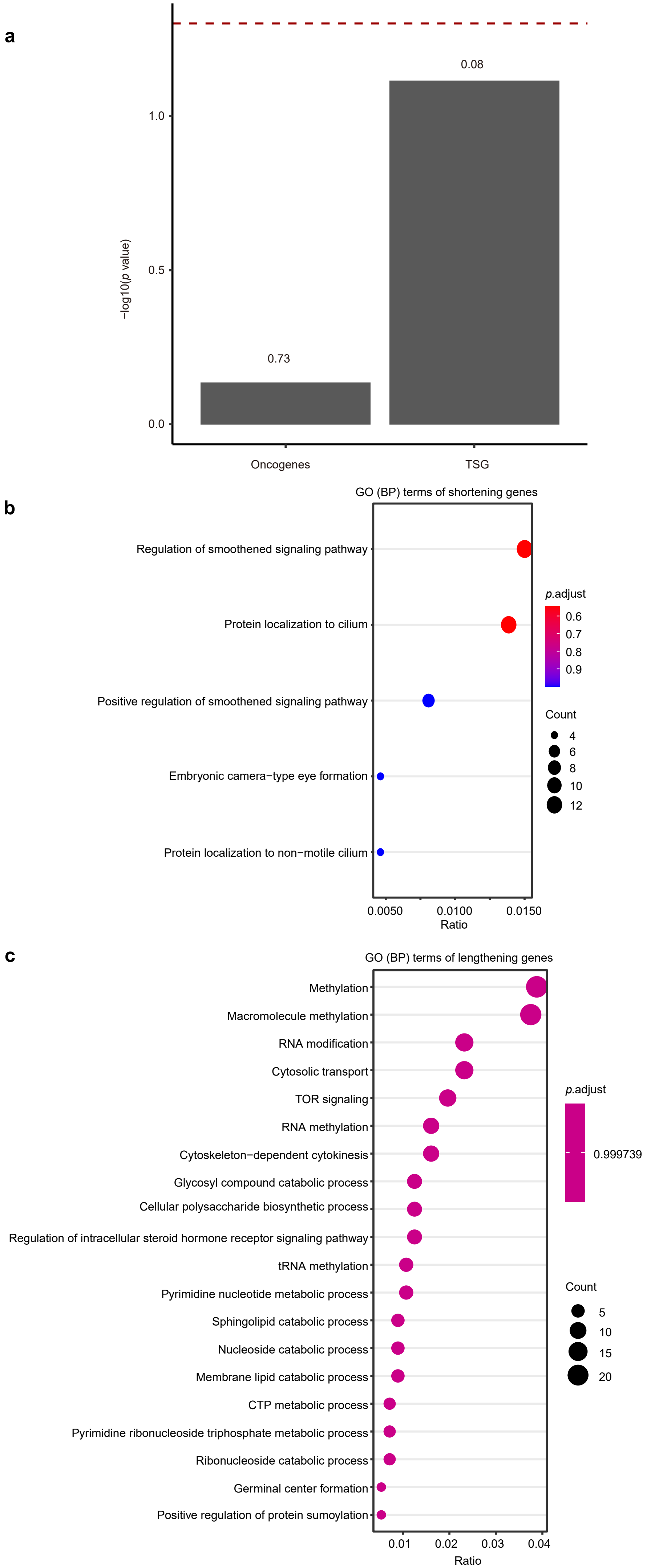

Supplementary Fig. S3 RBBP6 shortening and lengthening genes are not enriched in the OGs/TSGs-related datasets and pathways.

a The number of shortening and lengthening genes that overlap with the set of OGs/TSGs follows a hypergeometric distribution. We obtained the top 200 predicted OGs and TSGs from Tumor Suppressor and Oncogene (TUSON) Explorer. The y axis is the signed log10 p-values of the hypergeometric test p values. **b, c** Gene ontology (GO) term enrichment analysis for the RBBP6 shortening and lengthening genes (FDR ⩽ 0.05 and PDUI difference ⩾0.2).

Supplementary Fig. S4

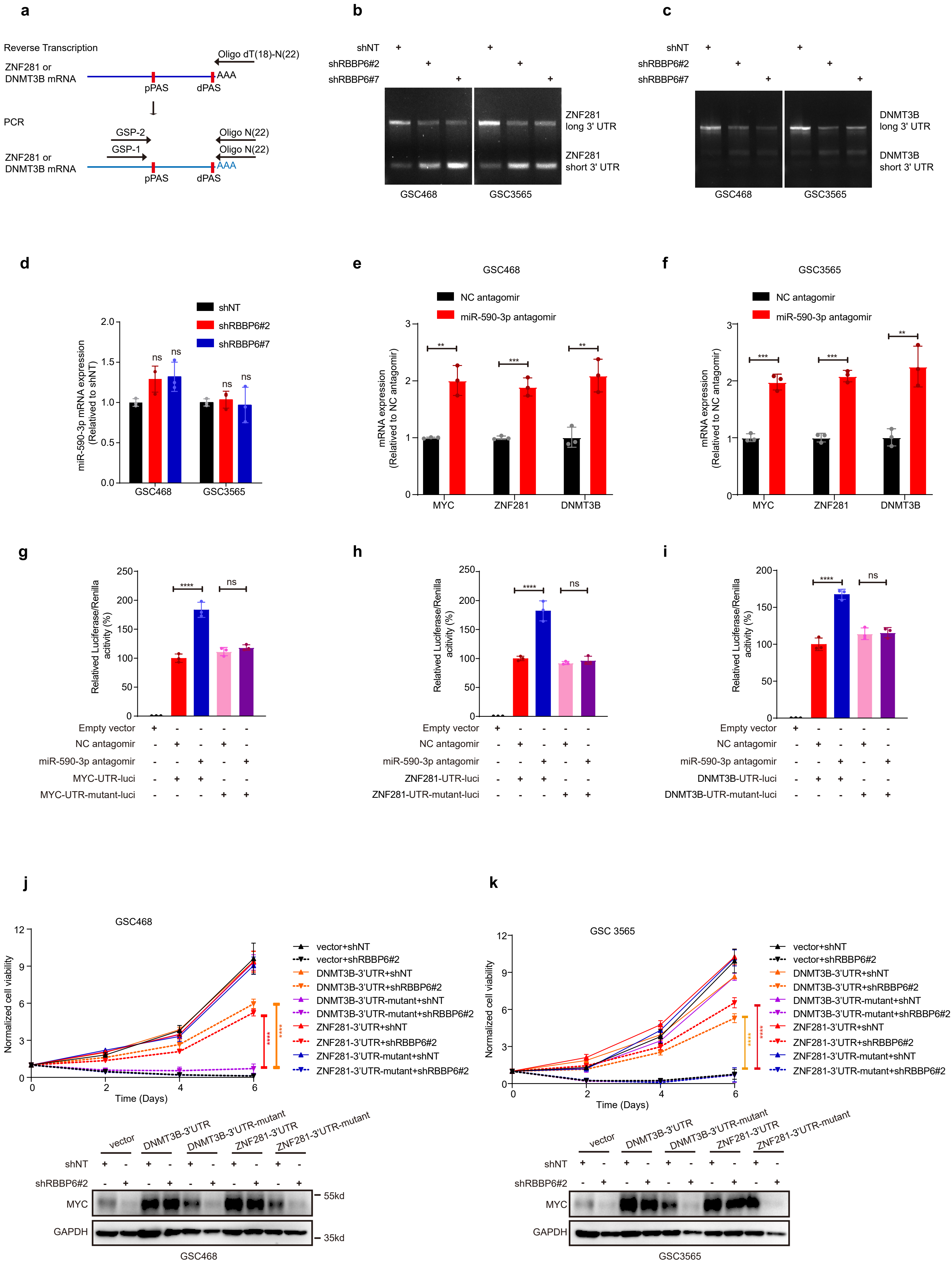

Supplementary Fig. S4 *MYC*, *ZNF281* and *DNMT3B* mRNAs are targets of miR-590-3p.

a-c 3'RACE was performed with GSP-1/2 primers (a) to evaluate 3'UTR of *ZNF281* and *DNMT3B* in GSCs (**b-c**). **d** The mRNA level of *miR-590-3p* in GSC468 and GSC3565 cells transduced separately with two *RBBP6*-targeting shRNAs or a nontargeting shRNA (shNT). Statistical significance was assessed using an ordinary one-way ANOVA with Dunnett's multiple comparisons test, ns, no significant; n = 3. **e** mRNA levels of *MYC*, *ZNF281* and *DNMT3B* in GSC468 cells transfected with miR-590-3p antagomir or NC antagomir. Statistical significance was assessed using a t test, ** p <0.01, *** p <0.001, n = 3. **f** mRNA levels of *MYC*, *ZNF281* and *DNMT3B* in GSC3565 cells transfected with miR-590-3p antagomir or NC antagomir. Statistical significance was assessed using a t test, ** p <0.01, *** p <0.001, n = 3. **g** Luciferase reporter assay showing miR-590-3p binding to the *MYC* 3'UTR. Renilla luciferase activity was used for luciferase activity normalization. Statistical significance was assessed using an ordinary one-way ANOVA with Dunnett's multiple comparisons test, **** p <0.0001, ns, no significant; n = 3. **h** The luciferase reporter assay showed miR-590-3p binding to the *ZNF281* 3'UTR. Renilla luciferase activity was used for luciferase activity normalization. Statistical significance was assessed using an ordinary one-way ANOVA with Dunnett's multiple comparisons test, **** p <0.0001, ns, no significant; n = 3. **i** The luciferase reporter assay showed miR-590-3p binding to the *DNMT3B* 3'UTR. Renilla luciferase activity was used for luciferase activity normalization. Statistical significance was assessed using an ordinary one-way ANOVA with Dunnett's multiple comparisons test, *** p <0.001, ns, no significant; n = 3. **j** (Top) Cell viability in GSC468-vector, GSC468-DNMT3B-3'UTR overexpressing, GSC468-DNMT3B-3'UTR-mutant overexpressing, GSC468-ZNF281-3'UTR overexpressing and GSC468-ZNF281-3'UTR mutant overexpressing cells following knockdown with a shRNA targeting *RBBP6* or a nontargeting shRNA (shNT). The error bars show the SDs. Two-way repeated measures ANOVA was used for statistical analysis with Sidak's multiple comparisons test, ****, p < 0.0001, n = 3. (Bottom) Western blot analysis of the GSC468 cell model from (Top). **k** (Top) Cell viability in GSC3565-vector, GSC3565-

DNMT3B-3'UTR overexpressing, GSC3565-DNMT3B-3'UTR-mutant overexpressing, GSC3565-ZNF281-3'UTR overexpressing and GSC3565-ZNF281-3'UTR mutant overexpressing cells following knockdown with a shRNA targeting *RBBP6* or a nontargeting shRNA (shNT). The error bars show the SDs. Two-way repeated measures ANOVA was used for statistical analysis with Sidak's multiple comparisons test, ****, $p < 0.0001$, $n = 3$. (Bottom) Western blot analysis of the GSC3565 cell model from (Top).

Supplementary Fig. S5

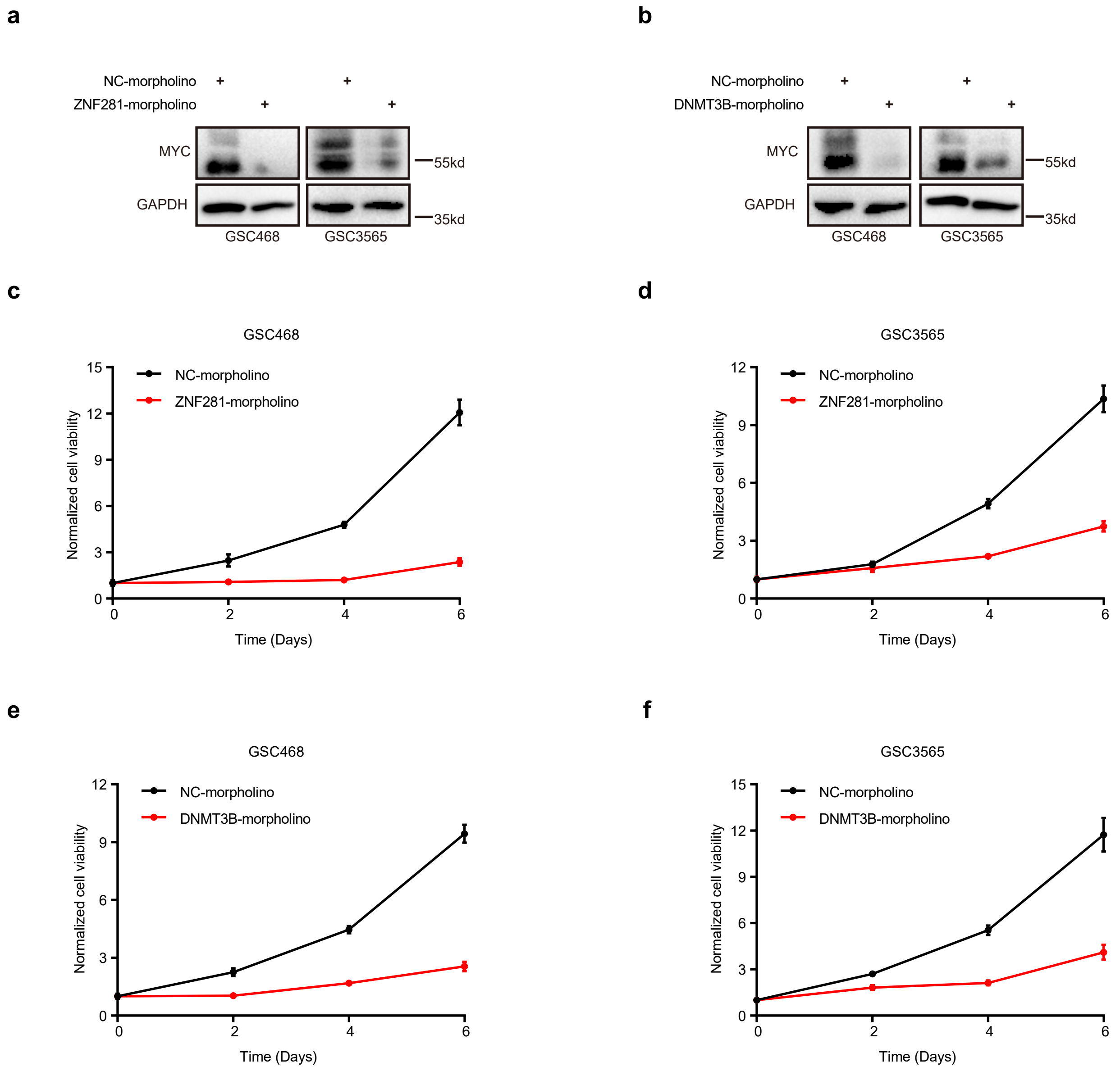

Supplementary Fig. S5 The blockade of miR-590-3p binding site on *DNMT3B* or *ZNF281* consequently inhibited the expression of MYC and impaired GSC proliferation.

a Western blot of MYC in GSC468 and GSC3565 cells transfected with NC-morpholino or ZNF281-morpholino. **b** Western blot of MYC in GSC468 and GSC3565 cells transfected with NC-morpholino or DNMT3B-morpholino. **c-d** Cell viability in the GSC468 cell model (**c**) and GSC3565 cell model (**d**) transfected with NC-morpholino or ZNF281-morpholino. Three technical replicates were used for each group. The error bars show the SDs. **e-f** Cell viability in the GSC468 cell model (**e**) and GSC3565 cell model (**f**) transfected with NC-morpholino or DNMT3B-morpholino. Three technical replicates were used for each group. The error bars show the SDs.

Supplementary Fig. S6

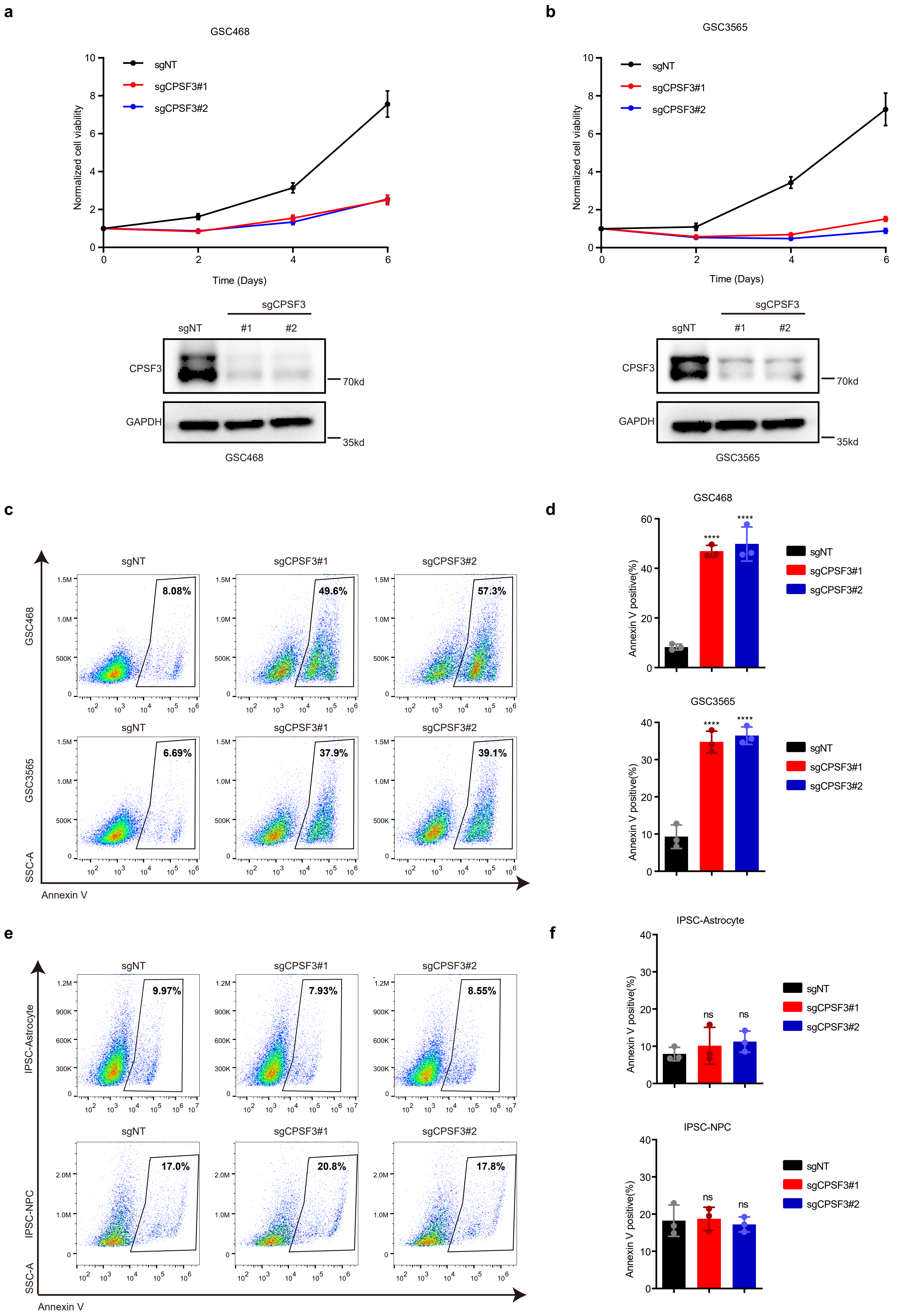

Supplementary Fig. S6 CPSF3 knockout led to GSC proliferation defect and GSC apoptosis.

a, b (Top) Cell viability in the GSC468 cell model (**a**) and GSC3565 cell model (**b**) following knockout with two sgRNAs targeting *CPSF3* or a nontargeting sgRNA (sgNT). Three technical replicates were used for each group. The error bars show the SDs. (Bottom) Western blot results from (Top). **c, d** Annexin V staining of GSC3565 and GSC468 cells transduced with two separate sgRNAs targeting *CPSF3* or a nontargeting sgRNA (sgNT). Three technical replicates were used for each condition. Quantification of Annexin-V staining using an ordinary one-way ANOVA with Dunnett's multiple comparisons test, **** $p < 0.0001$, $n = 3$. **e, f** Annexin V staining of IPSC-Astrocyte and IPSC-NPC cells transduced with two separate sgRNAs targeting *CPSF3* or a nontargeting sgRNA (sgNT). Three technical replicates were used for each condition. Quantification of Annexin-V staining using an ordinary one-way ANOVA with Dunnett's multiple comparisons test, ns, no significant; $n = 3$.

Supplementary Fig. S7

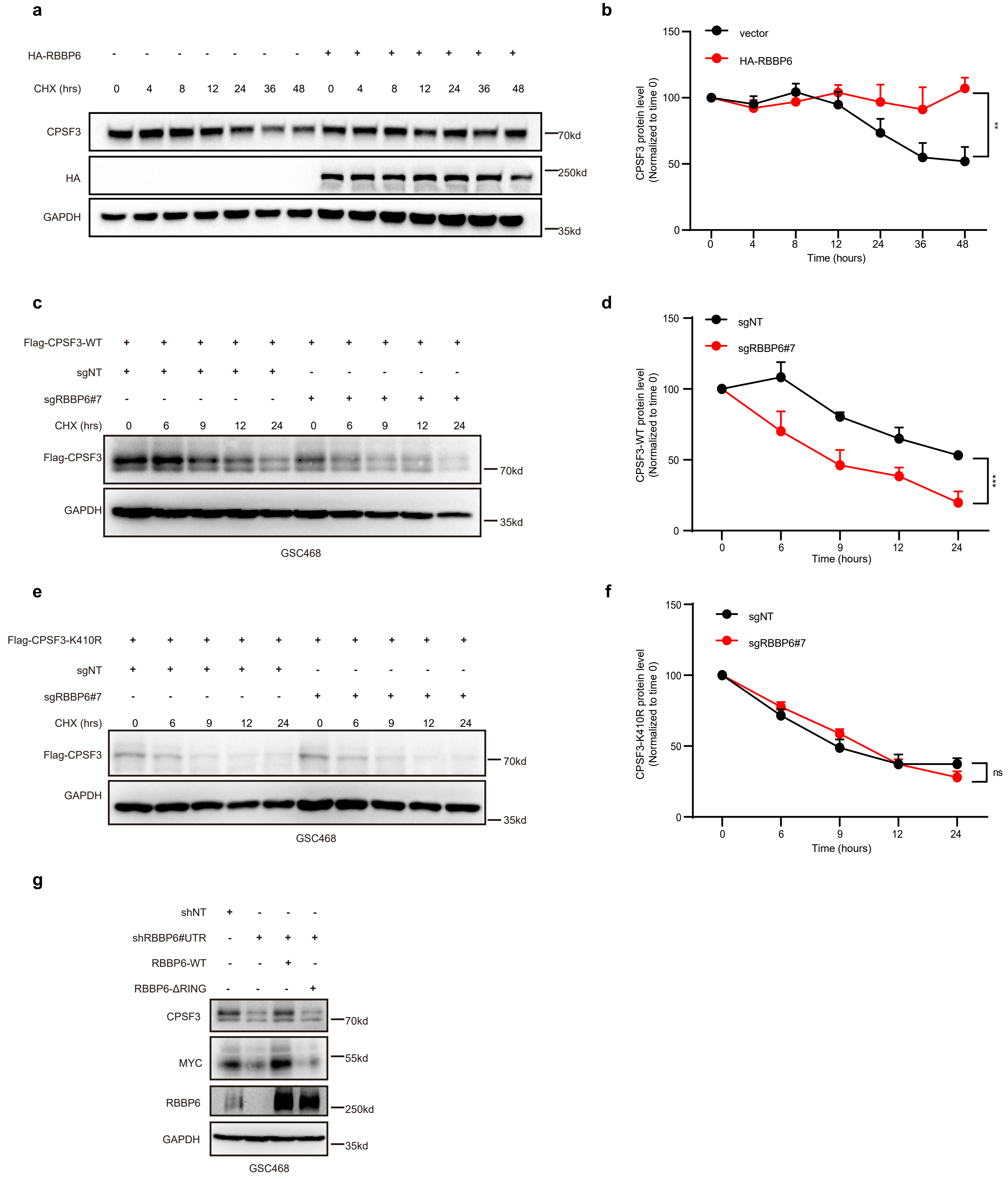

Supplementary Fig. S7 RBBP6 stabilized the CPSF3 protein level

a HEK293T cells transfected HA-RBBP6 or control vector followed by CHX (50 μg/ml) treatment for the indicated times were collected for IB. **b** Quantification of the results shown in (**a**). Two-way ANOVA was used for statistical analysis with Sidak's multiple comparisons test. **, $p < 0.01$, n = 3. **c** The GSC468-CPSF3-WT overexpression cells transduced with a sgRNA targeting RBBP6 or a nontargeting sgRNA (sgNT) followed by CHX (50 μg/ml) treatment for the indicated times were collected for IB. **d** Quantitation of the results shown in (**c**). Two-way ANOVA was used for statistical analysis with Sidak's multiple comparisons test. ***, $p < 0.001$, n = 3. **e** The GSC468-CPSF3-K410R overexpression cells transduced with a sgRNA targeting RBBP6 or a nontargeting sgRNA (sgNT) followed by CHX (50 μg/ml) treatment for the indicated times were collected for IB. **f** Quantitation of the results shown in (**e**). Two-way ANOVA was used for statistical analysis with Sidak's multiple comparisons test. ns, no significance; n = 3. **g** Western blot of MYC and CPSF3 in GSC468-vector, GSC468-RBBP6 overexpressing and GSC468-RBBP6-ΔRING overexpressing cells following knockdown with a shRNA targeting RBBP6.

Supplementary Fig. S8

a

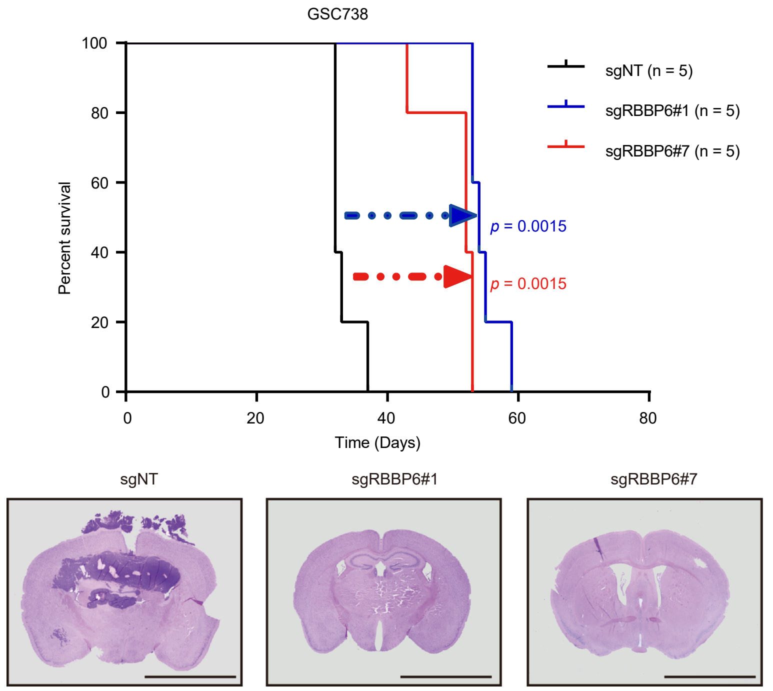

Scale bar: 5 mm

b

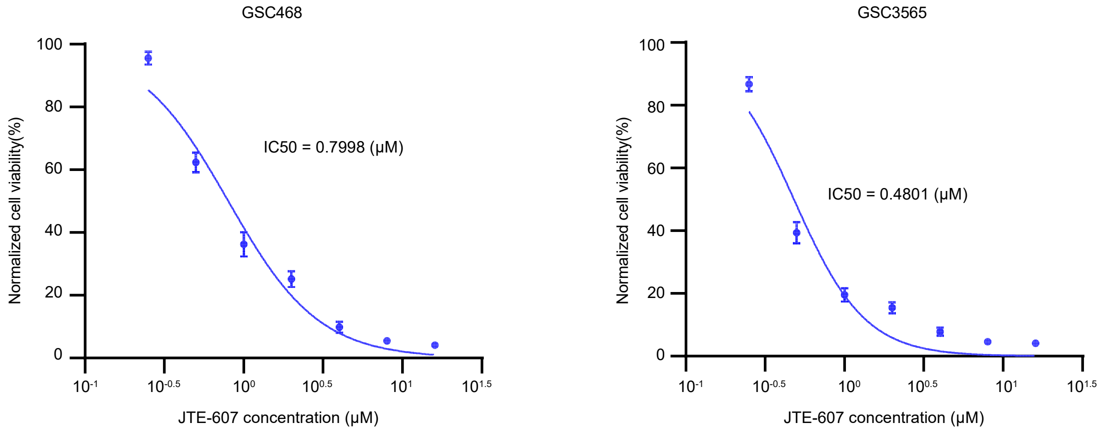

c

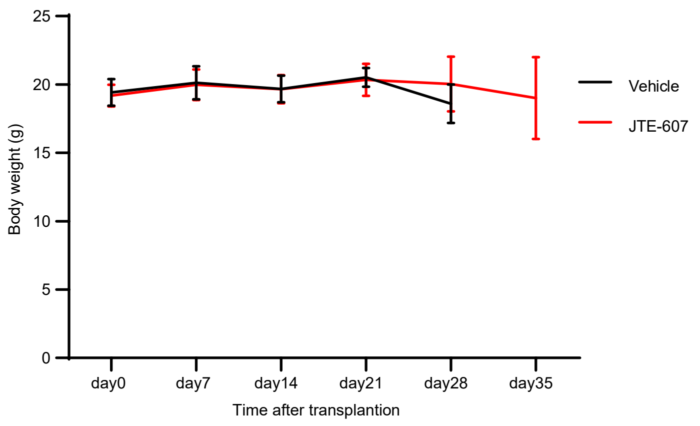

Supplementary Fig. S8 Targeting CPSF3 inhibited GSC proliferation.

a (Top) Kaplan–Meier survival curves of immunocompromised NSG mice bearing intracranial GSC738 cells transduced with sgNT, sgRBBP6#1, or sgRBBP6#7. (Bottom) Representative images of HE staining of mouse brains. Brains were isolated after the presentation of the first neurological sign in any cohort. Scale bar, 5 mm. Log-rank test was used for statistical analysis. **b** Dose–response curves of JTE-607 treatment in GSC468 (left) and GSC3565 (right) cell model. The data are presented as the means ± SDs. **c** The body weight of vehicle or JTE-607 treated mice.
